# Synthesis and Anti-Yeast Evaluation of Novel 2-Alkylthio-4-chloro-5-methyl-*N*-[imino-(1-oxo-(1*H*)-phthalazin-2-yl)methyl]benzenesulfonamide Derivatives

**DOI:** 10.3390/molecules190913704

**Published:** 2014-09-02

**Authors:** Jarosław Sławiński, Aneta Pogorzelska, Beata Żołnowska, Anna Kędzia, Marta Ziółkowska-Klinkosz, Ewa Kwapisz

**Affiliations:** 1 Department of Organic Chemistry, Medical University of Gdańsk, Al. Gen. J. Hallera 107, Gdańsk 80-416, Poland; E-Mails: anetapogorzelska@gumed.edu.pl (A.P.); zolnowska@gumed.edu.pl (B.Ż.); 2 Department of Oral Microbiology, Medical University of Gdańsk, ul. Do Studzienki 38, Gdańsk 80-227, Poland; E-Mails: anak@gumed.edu.pl (A.K.); martaz-k@gumed.edu.pl (M.Z.-K.); kwapisz@gumed.edu.pl (E.K.)

**Keywords:** sulfonamides, phthalazine, antifungal agents, structure-activity relationship, *Candida*

## Abstract

Pathogenic fungi are one of the main causes of hospital-related infections. Since conventional antifungals have become less effective because of the increasing fungal resistance to the standard drugs, the need for new agents is becoming urgent. Herein we report a synthesis of a series of novel *N*-[imino-(1-oxo-(1*H*)-phthalazin-2-yl)methyl]-benzenesulfonamide derivatives with *in vitro* activity against yeast-like fungi isolated from the oral cavity and respiratory tract of patients with candidiasis. These compounds were synthesized by the one-step or two-step reactions of 1-(2-alkylthiobenzensulfonyl)-2-aminoguanidines with the appropriate *ortho*-carbonyl benzoic acids. The biological study revealed that new derivatives have shown significant growth-inhibitory activity, superior or comparable, than those of the reference drug fluconazole. The most promising activities were observed against *Candida albicans*, with inhibition at least 1–3 (12.5%–37.5%) of the eight tested strains at the low MIC level of ≤6.2–25 µg/mL.

## 1. Introduction

Until recently, fungal infections were a major threat for patients with hematological disorders [1,2], but today there are a wide range of predisposing factors, such as immunosuppressive therapy, organ transplantation, corticosteroid therapy, solid tumors, AIDS, lymphoproliferating diseases, diabetes mellitus, the presence of indwelling intravenous catheters, prolonged antibiotic treatment, chronic renal failure, hemodialysis and intravenous hyperalimentation [3,4,5,6,7,8].

*Candida* spp. are the most common cause of mycoses. Among them *C. albicans* has been known as the most threatening pathogen, however, other, non-*albicans* species, including *C. glabrata*, *C. parapsilosis*, *C. tropicalis*, *C. krusei* and *C. dubliniensis* have also been reported as serious causes of fungal infections [4,9,10,11,12,13,14]. The high pathogenicity of *C. albicans* is due, *inter alia*, to its ability to form biofilms on different surfaces which is known as a serious factor for significantly increasing resistance to antifungal agents and protection from host defenses, the main reasons why fungal infections are frequently refractory to conventional therapy [15,16,17]. This occurrence, in turn, has very important implications for morbidity, mortality and health care costs in hospitals, as well in the community care. Moreover, the widespread use of antifungal drugs makes them become ineffective in the treatment of infections related to resistant pathogens [9,10,18,19,20,21,22].

**Figure 1:**
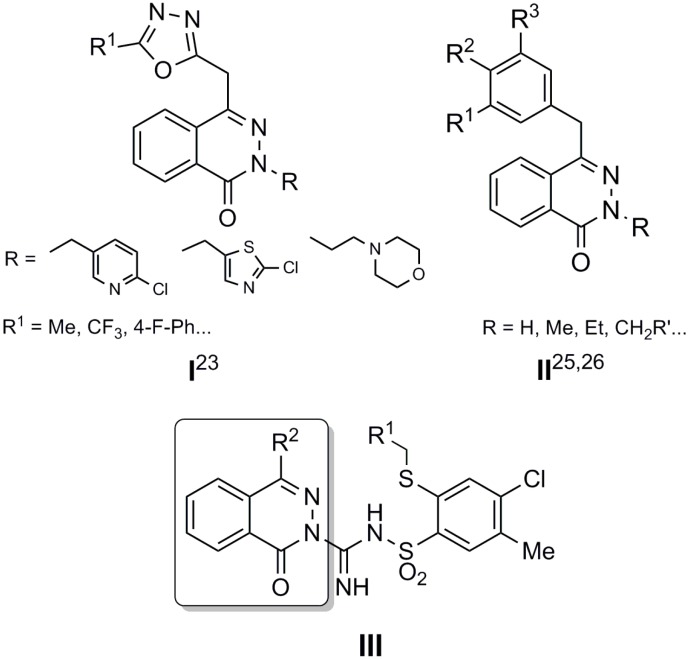
Phthalazine derivatives with antifungal activity [25,27,28,29].

Both the limited number of efficacious antifungal drugs and resistance to antifungal therapy are the reason for the search for new useful agents with unique mechanisms of action [22,23,24]. Evaluations of the antimicrobial activity of some phthalazine derivatives revealed that these compounds show potent antifungal activity (Figure 1, **I**, **II**) [25,26,27,28,29]. These findings encouraged us to synthesize a series of 2-alkylthio-4-chloro-5-methyl-*N*-[imino-(1-oxo-2(1*H*)-phthalazin-2-yl)]benzenesulfonamides (Figure 1, **III**) to investigate of their potential antifungal activity.

## 2. Results and Discussion

### 2.1. Chemistry

**Scheme 1:**
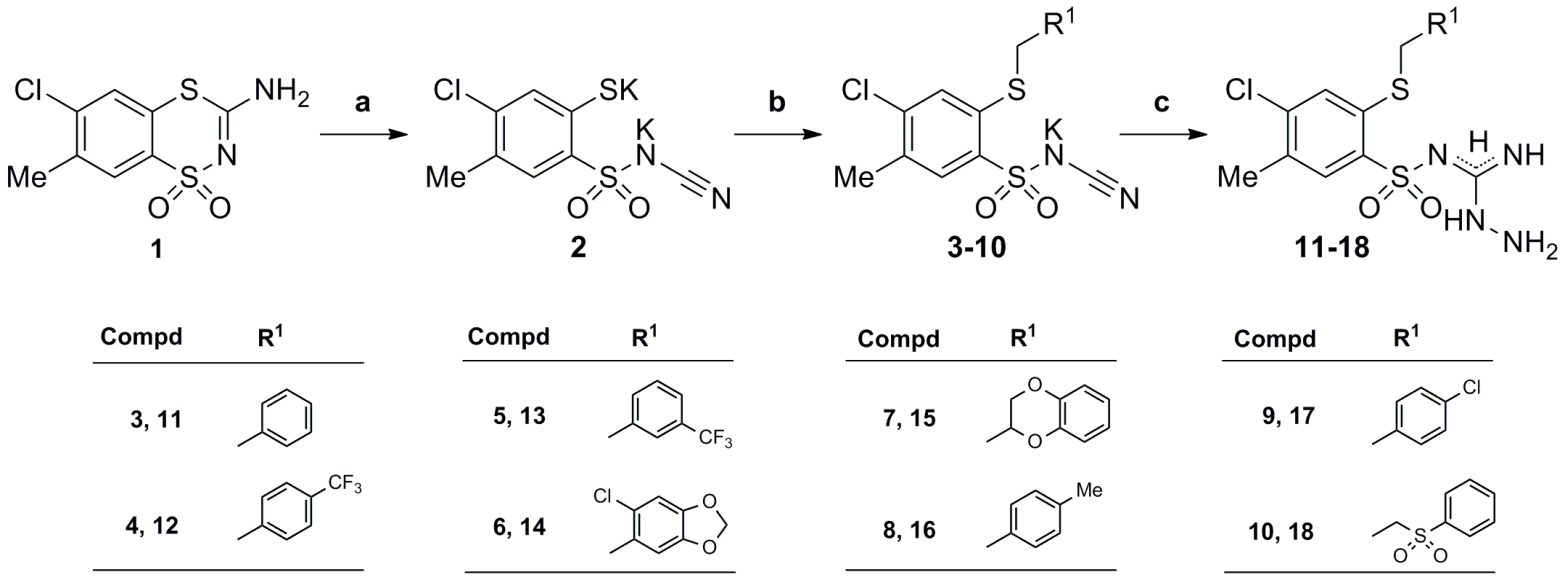
Facile three-step synthesis of the starting *N*-amino-*N'*-(2-alkylthio-4-chloro-5-methylbenzenesulfonyl)guanidine derivatives **11**–**18**.

As presented in Scheme 1 the novel *N*-(2-alkylthio-4-chloro-5-methylbenzenesulfonyl)cyanamide potassium salts **8**–**10** and *N*-amino-*N'*-(2-alkylthio-4-chloro-5-methylbenzenesulfonyl)guanidines **12**–**18** were obtained analogously to the already described methods in [30,31,32] for **8**–**10**, and [33] for **12**–**18**, respectively.

*Reagents and conditions*: (**a**) Excess K_2_CO_3_, THF, reflux, 24 h; (**b**) R^1^CH_2_Cl, ethanol or water, rt or 0 °C, 1–4 h; (**c**) NH_2_-NH_2_ × HCl, dry toluene, reflux, 3–10 h.

**Scheme 2:**
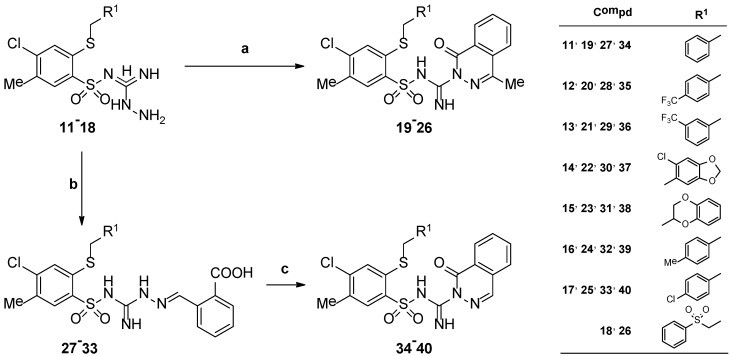
Synthesis of 2-alkylthio-4-chloro-5-methyl-*N*-[imino-(4-methyl-1-oxo-(1*H*)-phthalazin-2-yl)methyl]benzenesulfonamides **19**–**26**, *N*-(2-alkylthio-4-chloro-5-methyl-benzenesulfonyl)-2-(2-carboxybenzylidene)hydrazinecarboximidamides **27**–**33** and 2-alkylthio-4-chloro-5-methyl-*N*-[imino-(1-oxo-(1*H*)-phthalazin-2-yl)methyl]benzenesulfon-amides **34**–**40**.

The reaction of aminoguanidines **11**–**18** with the appropriate *ortho-*carbonyl substituted benzoic acids led to the novel 2-alkylthio-4-chloro-5-methyl-*N*-[imino-(4-methyl-1-oxo-(1*H*)-phthalazin-2-yl)methyl]benzenesulfonamides **19**–**26** or *N*-(2-alkylthio-4-chloro-5-methylbeneznesulfonyl)-2-(2-carboxybenzylidene)hydrazinecarboximidamides **27**–**33**, depending on the carbonyl group type as it shown in Scheme 2. Thus, treatment of aminoguanidines **11**–**18** with 2-acetylbenzoic acid resulted in the formation of the desired *N*-[imino-(4-methyl-1-oxo-(1*H*)-phthalazin-2-yl)methyl]benzenesulfonamide derivatives **19**–**26** in sufficient yields when the reaction mixture was refluxed in glacial acetic acid or, alternatively, in case of **25**, in dry 1,4-dioxane. In turn, treatment of the aminoguanidines **11**–**17** with 2-formylbenzoic acid furnished the appropriate non-cyclic *N*-substituted hydrazinecarboximidamide derivatives **27**–**33**, which could be easily converted to the expected 2-alkylthio-4-chloro-5-methyl-*N*-[imino-(1-oxo-(1*H*)-phthalazin-2-yl)methyl]benzenesulfon- amide derivatives **34**–**40** after heating in dry toluene in the presence of *p*-toluenesulfonic acid for 1–4.5 h. The structures of novel compounds were confirmed by elemental analyses (C, H, N) and spectroscopic data presented in the Experimental Section. The most characteristic absorption bands in IR spectra for compounds **19**–**40** were those derived from NH and C=O groups that appeared in the range of 3479–3208 cm^−1^ and 1683–1618 cm^−1^, respectively. In turn, the ^1^H-NMR spectra of a series of benzenesulfonamides **19**–**26** and **34**–**40** bearing 1-oxo-phthalazine moiety revealed multiplets at range 7.89–8.06 ppm attributable for protons H-5, H-6 and H-7 and doublet signals in the region of 8.16–8.33 for H-8 from the phthalazine ring, while its H-4 proton (**34**–**40**) gave resonance singlet signals at δ = 7.79–7.85 ppm.

*Reagents and conditions*: (**a**) 2-Acetylbenzoic acid (1 equiv), glacial acetic acid or 1,4-dioxane, reflux, 1–8 h; (**b**) 2-formylbenzoic acid (1 equiv), glacial acetic acid, reflux, 1–11 h; (**c**) *p*-toluene-sulfonic acid (0.3 equiv), toluene, reflux, 1–4.5 h.

### 2.2. Antifungal Activity

The fifteen newly synthesized compounds **19**–**26** and **34**–**40** have been tested *in vitro* for their antifungal activity against yeast-like fungi isolated from the oral cavity and respiratory tract of patients with candidiasis. Thirty-one yeast strains belonging to the genus: *Candida* (26 strains), *Geotrichum* (two strains), *Rhodotorula* (two strains) and *Saccharomyces* (one strain) were isolated from patients and used for testing. The ranges of MIC values obtained for each of the tested species are listed in Table 1. The investigated compounds showed meaningful or moderate activity against all tested pathogens, comparable or even higher than that of the reference drug fluconazole.

All the investigated compounds showed activity against *Candida albicans*. Most of them demonstrated the ability to inhibit at least four (50%) of the eight tested *C. albicans* strains at the MIC range of ≤6.2–100 µg/mL (Table 2). Furthermore, eight compounds (**22**–**24**, **26**, **34**, **36**, **37** and **40**) showed lower or comparable MIC values to fluconazole (Table 2).

**Table 1:** The range of MICs obtained for the synthesized compounds **19**–**26** and **34**–**40** and fluconazole against strains isolated from patients.

**Compound**	**MIC ^a^ (µg/mL)**										
	*Candida albicans* (8 strains)	*Candida glabrata* (4 strains)	*Candida guilliermondii* (2 strains)	*Candida krusei* (3 strains)	*Candida lusitaniae* (2 strains)	*Candida parapsilosis* (3 strains)	*Candida tropicalis* (3 strains)	*Candida utilis* (1 strain)	*Geotrichum candidum* (2 strains)	*Rhodothorula mucilaginosa* (2 strains)	*Saccharomyces cerevisiae* (1 strain)
**19**	50–200	*	*	*	100–200	*	*	100	*	25–50	*
**20**	100–200	*	25–100	*	*	50–200	100–200	100	100–200	*	*
**21**	100–200	*	50–100	*	100–200	50–200	50–200	50	*	*	*
**22**	12.5–200	*	50–100	*	100–200	100–200	100–200	*	*	*	*
**23**	25–200	*	25–100	*	*	50–200	50–200	*	*	*	*
**24**	25–200	100–200	*	50–200	*	*	*	*	*	≤6.2–12.5	*
**25**	50–200	*	12.5–100	*	*	12.5–200	12.5–200	50	100 ^b^	100 ^b^	*
**26**	25–200	*	100–200	100–200	100–200	*	*	100	*	≤6.2–50	*
**34**	25–200	*	25 ^b^	100–200	25–50	≤6.2–200	50–200	25	12.5–50	50–100	100
**35**	50–200	*	50–200	*	100–200	100–200	50–200	50	*	*	*
**36**	≤6.2–200	*	12.5–100	*	25–100	50–200	50–200	100	*	*	*
**37**	12.5–200	*	*	*	*	*	*	50	*	25–50	*
**38**	100–200	100–200	50–100	50–200	100–200	≤6.2–200	100–200	100	*	100–200	*
**39**	100–200	*	100 ^b^	*	100 ^b^	*	*	*	*	25–50	*
**40**	25–200	100–200	25–100	*	50–100	50–200	50–200	≤6.2	100 ^b^	*	100
**Fluconazole**	25–100	50–100	12.5–25	25–100	12.5–25	6.2–25	≥100	25	50–100	≥100	≥100

^a^ Minimum inhibitory concentration; ^b^ value obtained for all tested strains; * MIC value ≥ 200 µg/mL.

**Table 2:** Susceptibility of eight isolated strains of *Candida albicans* to the most active compounds.

Compd	Number of Susceptible Strains at MIC (µg/mL) of:				
	≤6.2	12.5	25	50	100
**19**				2	2
**22**		1	1		1
**23**			3		1
**24**			3	1	
**25**				3	3
**26**			1		3
**34**			2	1	4
**35**				3	1
**36**	1	1			3
**37**		1	1	2	1
**40**			1	3	3
**F**			1	1	6 ^a^

^a^ for fluconazole MIC ≥ 100 µg/mL; **F**—fluconazole (Fluka).

Most of the tested compounds showed potent antifungal activity in respect to *Candida guilliermondii*, *Candida tropicalis* and *Candida utilis*. The seven derivatives (**21**, **23**, **25**, **34**–**36** and **40**) displayed higher activity against *C.* *tropicalis* as compared with fluconazole (see Table 3). The inhibition of the growth of *C*. *guilliermondii* was comparable with reference for **25** and **36** (Table 3). The similar to fluconazole MIC values against *C. utilis* was noticed for **34** and **40** (see Table 1).

Among the tested compounds a good activity profile against *Rhodotorula mucilaginosa* was noticed for eight derivatives*.* Interestingly, six of them (compounds **19**, **24**, **26**, **34**, **37** and **39**) showed a significantly higher activity than those of fluconazole as was outlined in Table 3. Moreover, most of these compounds exhibited good selectivity against *R. mucilaginosa*.

The eight compounds displayed activity against *Candida parapsilosis* wherein the derivatives **34** and **38** reached an MIC ≤6.2 µg/mL for one (33.3%) of the tested strains, which is comparable to the value obtained for fluconazole (Table 3).

Low activity against *C. glabrata* and *C. krusei* has been observed. Inhibition of these species, which are inherently resistant to fluconazole, was noticed for compounds **24**, **38**, **40** and **24**, **26**, **34**, **38** respectively. Interestingly, derivatives **24** and **38** displayed good selectivity for the growth inhibition of *C. glabrata* and *C. krusei*.

The investigation of the structure-activity relationship (SAR) showed, in general, that the lack of a methyl group at position 4 of the 1-phthalazinone system enhanced the antifungal action of the synthesized compounds. In the series of 2-alkylthio-4-chloro-5-methyl-*N*-[imino-(4-methyl-1-oxo-(1*H*)-phthalazin-2-yl)methyl]benzenesulfonamides **19**–**26** having a methyl group in the 4-position (R^2^ = Me) of the 1-phthalazinone system, it can be seen that the insertion of a 4-chlorobenzylthio group (R^1^ = 4-ClPh, **25**), as well as bulky substituents (**22**–**24**, **26**) at the position 2 of the benzenesulfonamide usually resulted in either low MIC or good selectivity against tested fungi.

The structure activity relationship analysis for the series of 2-alkylthio-4-chloro-5-methyl-*N*-[imino-(1-oxo-(1*H*)-phthalazin-2-yl)methyl]benzenesulfonamides **34**–**40** revealed that the presence of the benzylthio group (R^1^ = Ph, **34**) at the position 2 of benzenesulfonamide scaffold leads to the best antifungal activity. Moreover, the substitution pattern of the phenyl ring at positions 3 and 4 in the benzylthio group appears to be an important factor to affect their antifungal activity. A good activity profile was found for compound **36** bearing a 3-(trifluoromethyl)benzylthio group (R^1^ = 3-CF_3_Ph) in the position 2 of the benzenesulfonamide scaffold. Similar findings were also observed for compound **40** substituted in this position by a 4-chlorobenzylthio group (R^1^ = 4-ClPh). On the other hand, the replacement of the chlorine atom (*i.e*., compound **40**) in the position 4 of the phenyl substituent by a more bulky substituent such as a trifluoromethyl (**35**) or methyl (**39**) group appears to have an adverse effect on the tested activity. Insertion of these groups has led to a significant increase in the MIC values.

**Table 3:** Compounds having similar or higher activity than fluconazole.

Organism (no. tested) and Compd	Number of Susceptible Strains at MIC (µg/mL) of:				
	≤6.2	12.5	25	50	100
*Candida tropicalis* (three strains)					
**21**				1	
**23**				1	
**25**		1			
**34**				1	
**35**				1	
**36**				1	
**40**				1	
**F**					3 ^a^
*Candida guilliermondii* (two strains)					
**25**		1			
**35**		1			
**F**		1	1		
*Candida parapsilosis* (three strains)					
**34**	1				
**38**	1				
**F**	1	1	1		
*Rhodothorula mucilaginosa* (two strains)					
**19**			1	1	
**24**	1	1			
**26**	1			1	
**34**				1	1
**37**			1	1	
**39**			1	1	
**F**					2 ^a^

^a^ for fluconazole MIC ≥ 100 µg/mL; **F**—fluconazole (Fluka).

Decreased antifungal activity was also observed if the benzylthio group at the position 2 of benzenesulfonamide (compound **34**) was replaced by larger substituents such as 6-chlorobenzo[*d*]-[1,3]dioxol-5-ylmethylthio (**37**) or 2,3-dihydrobenzo[*b*][1,4]-dioxan-2-ylmethylthio (**38**). The activity of compound **37** has been limited only to three species however, the obtained MICs against sensitive strains were comparable or higher than those of reference or compounds **34**, **36** and **40** having the best antifungal properties. The derivative **38**, in turn, showed relatively poor MIC as compared to both the most active derivatives (**34**, **36** and **40**) and fluconazole.

## 3. Experimental Section

### 3.1. General Procedures

The melting points were determined on a Boethius PHMK apparatus and are uncorrected. Infrared (IR) spectra were recorded on a Thermo Mattson Satellite FTIR spectrophotometer. The NMR spectra was carried out on a Varian Gemini 200 apparatus at 200 MHz (^1^H-NMR) and 50 MHz (^13^C-NMR) or on a Varian Unity 500 Plus apparatus at 500 MHz (^1^H-NMR) and 125 MHz (^13^C-NMR). Chemical shifts are expressed as δ values in parts per million (ppm) relative to TMS as internal standard. Spectra were acquired in deuterated dimethylsulfoxide (DMSO-*d_6_*). The results of elemental analyses for C, H and N were in agreement with the theoretical values within ±0.4% range. The commercially unavailable substrates were obtained according to the following methods described previously: *N*-(2-benzylthio-4-chloro-5-methylbenzenesulfonyl)cyanamide potassium salt (**3**) [30], *N*-[4-chloro-5-methyl-2-(4-trifluoromethylbenzylthio)benzenesulfonyl]cyanamide potassium salt (**4**) [31], *N*-[4-chloro-5-methyl-2-(3-trifluoromethylbenzylthio)benzenesulfonyl]cyanamide potassium salt (**5**) [32], *N*-[4-chloro-2-(6-chlorobenzo[d][1,3]dioxol-5-ylmethylthio)-5-methylbenzenesulfonyl]cyanamide potassium salt (**6**) [32], *N*-[4-chloro-2-(2,3-dihydrobenzo[b][1,4]dioxan-2-ylmethylthio)-5-methyl- benzenesulfonyl]cyanamide potassium salt (**7**) [31], 1-amino-2-(2-benzylthio-4-chloro-5-methyl-benzenesulfonyl)guanidine (**11**) [33].

### 3.2. Synthesis

#### 3.2.1. General Procedure for the Preparation of *N*-(2-Alkylthio-4-chloro-5-methylbenzenesulfonyl)-cyanamide Potassium Salts **8**–**10**

A mixture of **2** (3 mmol) and the appropriate alkyl chloride (3.3 mmol) in water (9 mL) was stirred at 0 °C for 1–4 h. The solid was filtered off and crystallized from ethanol. In this manner the following potassium salts were obtained.

*N-[4-Chloro-5-methyl-2-(4-methylbenzylthio)benzenesulfonyl]cyanamide potassium salt* (**8**). Starting from **2** (1.017 g) and 4-methylbenzyl chloride (0.44 mL) in water with stirring for 1 h, 1.125 g (92%) of the title compound **8** was obtained, mp 198–200 °C; IR (KBr) ν_max_ 2922 (C-H), 2174 (C≡N), 1343, 1142 (SO_2_) cm^−1^; ^1^H-NMR (200 MHz, DMSO-*d_6_*) *δ* 2.28 (s, 3H, CH_3_), 2.32 (s, 3H, CH_3_), 4.2 (s, 2H, SCH_2_), 7.12–7.16 (d, *J* = 8.0 Hz, 2H, arom.), 7.3–7.34 (d, *J* = 7.9 Hz, 2H, arom.), 7.38 (s, 1H, H-3), 7.74 (s, 1H, H-6); anal. C 47.28, H 3.33, N 6.75% calcd for C_16_H_14_ClKN_2_O_2_S_2_, C 47.45, H 3.48, N 6.93%.

*N-[4-Chloro-2-(4-chlorobenzylthio)-5-methylbenzenesulfonyl]cyanamide potassium salt* (**9**). Starting from **2** (1.017 g) and 4-chlorobenzyl chloride (0.531 g) in water with stirring for 4 h, 1.253 g (98%) of the title compound **9** was obtained, mp 231–233 °C; IR (KBr) ν_max_ 2920 (C-H), 2175 (C≡N), 1342, 1143 (SO_2_) cm^−1^; ^1^H-NMR (200 MHz, DMSO-*d_6_*) *δ* 2.3 (s, 3H, CH_3_), 4.28 (s, 2H, SCH_2_), 7.34–7.50 (m, 5H, arom.), 7.73 (s, 1H, H-6); anal. C 42.33, H 2.58, N 6.55% calcd for C_15_H_11_Cl_2_KN_2_O_2_S_2_, C 42.35, H 2.61, N 6.59%.

*N-{4-Chloro-5-methyl-2-[(2-phenylsulfonyl)ethylthio]benzenesulfonyl}cyanamide potassium salt* (**10**). Starting from **2** (1.017 g) and 2-(phenylsulfonyl)ethyl chloride (0.675 g) in water with stirring for 1.5 h, 1.346 g (87%) of the title compound **10** was obtained, mp 126–129 °C; IR (KBr) ν_max_ 2922 (C-H), 2179 (C≡N), 1343, 1151 (SO_2_) cm^−1^; ^1^H-NMR (200 MHz, DMSO-*d_6_*) *δ* 2.31 (s, 3H, CH_3_), 3.12–3.19 (m, 2H, CH_2_S), 3.35–3.62 (m, 2H, CH_2_SO_2_), 7.26 (s, 1H, H-3), 7.63–7.7.81 (m, 4H, arom.), 7.92–7.96 (m, 2H, arom.); anal. C 40.90, H 2.96, N 5.90% calcd for C_16_H_14_ClKN_2_O_4_S_3_, C 40.97, H 3.01, N 5.97%.

#### 3.2.2. General Procedure for the Preparation of 1-Amino-2-(2-alkylthio-4-chloro-5-methylbenzene- sulfonyl)guanidines **12**–**18**

To a suspension of the appropriate *N*-(2-alkylthio-4-chloro-5-methylbenzenesulfonyl)cyanamide potassium salt **4**–**10** (3 mmol) in dry toluene (2.5–22.5 mL) hydrazine monohydrochloride (3 mmol) was added. A reaction mixture was stirred at reflux for 3–10 h, and left overnight at 0 °C. The precipitate was filtered off and dried, then treated with water (40 mL). After vigorously stirring for 30 min the precipitate was collected by filtration and dried. In this manner the following aminoguanidines were obtained:

*1-Amino-2-[4-chloro-5-methyl-2-(4-trifluoromethylbenzylthio)benzenesulfonyl]guanidine* (**12**). From **4** (1.377 g) in dry toluene (3 mL) with stirring for 4.5 h, 0.750 g (55%) of the title compound **12** was obtained, mp 210–212 °C; IR (KBr) ν_max_ 3482, 3332 (NH_2_, NH), 2959 (C-H), 1324, 1129 (SO_2_) cm^−1^; ^1^H-NMR (200 MHz, DMSO-*d_6_*) *δ* 2.28 (s, 3H, CH_3_), 4.40 (s, 2H, SCH_2_), 4.52 (s, 2H, CNH_2_), 7.00 (s, 2H, NNH_2_), 7.41 (s, 1H, H-3), 7.63 (d, 2H, arom.), 7.68 (d, 2H, arom.), 7.80 (s, 1H, H-6); anal. C 42.41, H 3.53, N 12.32% calcd for C_16_H_16_ClF_3_N_4_O_2_S_2_, C 42.43, H 3.56, N 12.37%.

*1-Amino-2-[4-chloro-5-methyl-2-(3-trifluoromethylbenzylthio)benzenesulfonyl]guanidine* (**13**). From **5** (1.377 g) in dry toluene (9 mL) with stirring for 3 h, 1.214 g (89%) of the title compound **13** was obtained, mp 154–155 °C; IR (KBr) ν_max_ 3437, 3333 (NH_2_, NH), 2924, 2854 (C-H), 1330, 1126 (SO_2_) cm^−1^; ^1^H-NMR (200 MHz, DMSO-*d_6_*) *δ* 2.29 (s, 3H, CH_3_), 4.41 (s, 2H, SCH_2_), 4.51 (s, 2H, CNH_2_), 6.98 (s, 2H, NNH_2_), 7.42 (s, 1H, H-3), 7.56–7.77 (m, 4H, arom.), 7.82 (s, 1H, H-6), 8.44 (s, 1H, NH); anal. C 42.38, H 3.49, N 12.30%, calcd for C_16_H_16_ClF_3_N_4_O_2_S_2_, C 42.43, H 3.56, N 12.37%.

*1-Amino-2-[4-chloro-2-(6-chlorobenzo[d][1,3]dioxol-5-ylmethylthio)-5-methylbenzenesulfonyl]-guanidine* (**14**). From **6** (1.401 g) in dry toluene (2.5 mL) with stirring for 4 h, 1.167 g (84%) of the title compound **14** was obtained, mp 245–250 °C; IR (KBr) ν_max_ 3476, 3366 (NH_2_, NH), 2957, 2905 (C-H), 1343, 1131 (SO_2_) cm^−1^; ^1^H-NMR (200 MHz, DMSO-*d_6_*) *δ* 2.32 (s, 3H, CH_3_), 4.24 (s, 2H, SCH_2_), 4.49 (s, 2H, CNH_2_), 6.08 (s, 2H, OCH_2_O), 6.98 (s, 2H, NNH_2_), 7.08 (s, 1H, arom.), 7.12 (s, 1H, arom.), 7.38 (s, 1H, H-3), 7.84 (s, 1H, H-6), 8.43 (s, 1H, NH); anal. C 41.45, H 3.49, N 12.10% calcd for C_16_H_16_Cl_2_N_4_O_4_S_2_, C 41.47, H 3.48, N 12.09%.

*1-Amino-2-[4-chloro-2-(2,3-dihydrobenzo[b][1,4]-dioxan-2-ylmethylthio)-5-methylbenzenesulfonyl]-guanidine* (**15**). From **7** (1.347 g) in dry toluene (3 mL) with stirring for 4 h, 1.157 g (87%) of the title compound **15** was obtained, mp 171–175 °C; IR (KBr) ν_max_ 3441, 3335, 3223 (NH_2_, NH), 2924, 2854 (C-H), 1344, 1132 (SO_2_) cm^−1^; ^1^H-NMR (200 MHz, DMSO-*d_6_*) *δ* 2.30 (s, 3H, CH_3_), 3.30 (s, 2H, SCH_2_), 4.01–4.11 (m, 1H, CHO), 4.30–4.40 (m, 2H, CH_2_O), 4.50 (s, 2H, CNH_2_), 6.80 (s, 4H, arom.), 7.00 (s, 2H, NNH_2_), 7.60 (s, 1H, H-3), 7.80 (s, 1H, H-6), 8.40 (s, 1H, NH); anal. C 45.99, H 4.31, N 12.63% calcd for C_17_H_19_ClN_4_O_4_S_2_, C 46.10, H 4.32, N 12.65%.

*1-Amino-2-[4-chloro-5-methyl-2-(4-methylbenzylthio)benzenesulfonyl]guanidine* (**16**). From **8** (1.215 g) in dry toluene (2.5 mL) with stirring for 4 h, 0.965 g (81%) of the title compound **16** was obtained, mp 200–202 °C; IR (KBr) ν_max_ 3472, 3364, 3342 (NH_2_, NH), 2958, 2924 (C-H), 1339, 1135 (SO_2_) cm^−1^; ^1^H-NMR (200 MHz, DMSO-*d_6_*) *δ* 2.25 (s, 3H, CH_3_), 2.35 (s, 3H, CH_3_), 4.25 (s, 2H, SCH_2_), 4.50 (s, 2H, CNH_2_), 7.00 (s, 2H, NNH_2_), 7.10–7.20 (d, *J* = 8.0 Hz, 2H, arom.), 7.28–7.36 (d, *J* = 8.0 Hz, 2H, arom.), 7.42 (s, 1H, H-3), 7.80 (s, 1H, H-6), 8.40 (s, 1H, NH); anal. C 48.14, H 4.75, N 14.00% calcd for C_16_H_19_ClN_4_O_2_S_2_, C 48.17, H 4.80, N 14.04%.

*1-Amino-2-[4-chloro-2-(4-chlorobenzylthio)-5-methylbenzenesulfonyl]guanidine* (**17**). From **9** (1.277 g) in dry toluene (18 mL) with stirring for 10 h, 1.070 g (85%) of the title compound **17** was obtained, mp 200–203 °C; IR (KBr) ν_max_ 3476, 3359 (NH_2_, NH), 2961, (C-H), 1342, 1127 (SO_2_) cm^−1^; ^1^H-NMR (200 MHz, DMSO-*d_6_*) *δ* 2.29 (s, 3H, CH_3_), 4.30 (s, 2H, SCH_2_), 4.51 (s, 2H, CNH_2_), 6.98 (s, 2H, NNH_2_), 7.34–7.50 (m, 5H, arom.), 7.80 (s, 1H, H-6), 8.42 (s, 1H, NH); anal. C 42.92, H 3.82, N 13.34% calcd for C_15_H_16_Cl_2_N_4_O_2_S_2_, C 42.96, H 3.85, N 13.36%.

*1-Amino-2-{4-chloro-5-methyl-2-[(2-phenylsulfonyl)ethylthio]benzenesulfonyl}guanidine* (**18**). From **10** (1.407 g) in dry toluene (16.5 mL) with stirring for 4 h, 1.361 g (98%) of the title compound **18** was obtained, mp 183–185 °C; IR (KBr) ν_max_ 3455, 3349 (NH_2_, NH), 2923, 2853 (C-H), 1314, 1144 (SO_2_) cm^−1^; ^1^H-NMR (200 MHz, DMSO-*d_6_*) *δ* 2.30 (s, 3H, CH_3_), 3.10–3.25 (m, 2H, SCH_2_), 3.50–3.65 (m, 2H, CH_2_SO_2_), 4.50 (s, 2H, CNH_2_), 6.97 (s, 2H, NNH_2_), 7.30 (s, 1H, H-3), 7.62–7.86 (m, 4H, arom.), 7.90–8.00 (m, 2H, arom.), 8.40 (s, 1H, NH); anal. C 41.49, H 4.13, N 12.05% calcd for C_16_H_19_ClN_4_O_4_S_3_, C 41.51, H 4.14, N 12.10%.

#### 3.2.3. General Procedure for the Preparation of 2-Alkylthio-4-chloro-5-methyl-*N*-[imino-(4-methyl-1-oxo-(1*H*)-phthalazin-2-yl)methyl]benzenesulfonamides **19**–**26**

To a suspension of the appropriate 1-amino-2-(2-alkylthio-4-chloro-5-methylbenzenesulfonyl)guanidine **11**–**18** (0.5 mmol) in glacial acetic acid (**19**–**22**, **24**–**26**—1 mL) or 1,4-dioxane (**23**—2.2 mL) 2-acetylbenzoic acid (0.082 g, 0.5 mmol) was added. A reaction mixture was stirred at reflux for 1–8 h, the precipitate was filtered off, dried and crystallized from acetonitrile (**19**, **24**, **26**), ethanol (**20**–**21**, **23**, **25**) or benzene (**22**). In this manner the following compounds were obtained:

*2-Benzylthio-4-chloro-5-methyl-N-[imino-(4-methyl-1-oxo-(1H)-phthalazin-2-yl)methyl]benzene-sulfonamide* (**19**). Starting from **11** (0.192 g) with stirring for 5 h, 0.110 g (51%) of the title compound **19** was obtained, mp 170–175 °C; IR (KBr) ν_max_ 3330, 3210 (NH), 2923, 2853 (C-H), 1664 (C=O), 1638 (NH), 1343, 1145 (SO_2_) cm^−1^; ^1^H-NMR (200 MHz, DMSO-*d_6_*) *δ* 2.20 (s, 3H, CH_3_Ph), 2.48 (s, 3H, CH_3_), 4.36 (s, 2H, SCH_2_), 7.25–7.35 (m, 3H, arom.), 7.44–7.48 (m, 2H, arom.), 7.51 (s, 1H, H-3), 7.77 (s, 1H, H-6), 7.91–8.05 (m, 3H, H-5, H-6, H-7 phthalazine), 8.29–8.33 (d, *J* = 7.8 Hz , 1H, H-8 phthalazine), 8.95 (s, 1H, C=NH), 9.29 (s, 1H, SO_2_NH); anal. C 56.17, H 4.16, N 11.05% calcd for C_24_H_21_ClN_4_O_3_S_2_, C 56.19, H 4.13, N 10.92%.

*4-Chloro-5-methyl-2-(4-trifluoromethylbenzylthio)-N-[imino-(4-methyl-1-oxo-(1H)-phthalazin-2-yl)-methyl]benzenesulfonamide* (**20**). Starting from **12** (0.209 g) with stirring for 1 h, 0.160 g (53%) of the title compound **20** was obtained, mp 169–174 °C; IR (KBr) ν_max_ 3331 (NH), 2923, 2853 (C-H), 1664 (C=O), 1642 (NH), 1528, 1447 (C=C, C=N), 1324, 1146 (SO_2_) cm^−1^; ^1^H-NMR (500 MHz, DMSO-*d_6_*) *δ* 2.15 (s, 3H, CH_3_Ph), 2.41 (s, 3H, CH_3_), 4.44 (s, 2H, SCH_2_), 7.48 (s, 1H, H-3), 7.57–7.59 (d, *J* = 7.5 Hz, 2H, arom.), 7.63–7.65 (d, *J* = 7.5 Hz, 2H, arom.), 7.72 (s, 1H, H-6), 7.90–8.02 (m, 3H, H-5, H-6, H-7 phthalazine), 8.26–8.27 (d, *J* = 7.8 Hz, 1H, H-8 phthalazine), 8.93 (s, 1H, C=NH), 9.29 (s, 1H, SO_2_NH); ^13^C-NMR (125 MHz, DMSO-*d_6_*) *δ* 18.4, 18.9, 35.9, 125.1, 126.1, 126.4, 126.9, 128.7, 129.2, 129.9, 130.8, 132.4, 132.5, 134.5, 135.4, 137.3, 138.4, 141.5, 144.2, 154.4, 157.2; anal. C 51.66, H 3.48, N 9.65% calcd for C_25_H_20_ClF_3_N_4_O_3_S_2_, C 51.68, H 3.47, N 9.64%.

*4-Chloro-5-methyl-2-(3-trifluoromethylbenzylthio)-N-[imino-(4-methyl-1-oxo-(1H)-phthalazin-2-yl)methyl]benzenesulfonamide* (**21**). Starting from **13** (0.209 g) with stirring for 1.5 h, 0.125 g (43%) of the title compound **21** was obtained, mp 145–149 °C; IR (KBr) ν_max_ 3333, 3208 (NH), 2923 (C-H), 1665 (C=O), 1639 (NH), 1526, 1449 (C=C, C=N), 1331, 1145 (SO_2_) cm^−1^; ^1^H-NMR (500 MHz, DMSO-*d_6_*) *δ* 2.16 (s, 3H, CH_3_Ph), 2.42 (s, 3H, CH_3_), 4.44 (s, 2H, SCH_2_), 7.47–7.50 (m, 2H, H-3, arom.), 7.57–7.59 (m, 2H, arom.), 7.76–7.78 (m, 3H, H-6, arom.), 7.89–8.01 (m, 3H, H-5, H-6, H-7 phthalazine), 8.25–8.27 (d, *J* = 7.8 Hz, 1H, H-8 phthalazine), 8.93 (s, 1H, C=NH), 9.28 (s, 1H, SO_2_NH); ^13^C-NMR (125 MHz, DMSO-*d_6_*) *δ* 19.0, 19.5, 36.7, 124.6, 124.7, 126.5, 126.8, 127.1, 127.6, 129.7, 129.8, 129.9, 130.2, 131.5, 133.1, 133.3, 133.9, 135.1, 136.0, 137.9, 138.7, 139.1, 144.9, 155.1, 157.9; anal. C 51.63, H 3.44, N 9.59% calcd for C_25_H_20_ClF_3_N_4_O_3_S_2_, C 51.68, H 3.47, N 9.64%.

*4-Chloro-2-(6-chlorobenzo[d][1,3]dioxol-5-ylmethylthio)-5-methyl-N-[imino-(4-methyl-1-oxo-(1H)-phthalazin-2-yl)methyl]benzenesulfonamide* (**22**). Starting from **14** (0.231 g) with stirring for 8 h, 0.127 g (43%) of the title compound **22** was obtained, mp 159–162 °C; IR (KBr) ν_max_ 3414, 3333 (NH), 2920 (C-H), 1662 (C=O), 1639 (NH), 1527, 1804 (C=C, C=N), 1345, 1145 (SO_2_) cm^−1^; ^1^H-NMR (200 MHz, DMSO-*d_6_*) *δ* 2.20 (s, 3H, CH_3_Ph), 2.47 (s, 3H, CH_3_), 4.30 (s, 2H, SCH_2_), 6.01 (s, 2H, OCH_2_O), 7.07 (s, 1H, arom.), 7.10 (s, 1H, arom.), 7.44 (s, 1H, H-3), 7.78 (s, 1H, H-6), 7.92–8.03 (m, 3H, H-5, H-6, H-7 phthalazine), 8.26–8.30 (d, *J* = 7.8 Hz, 1H, H-8 phthalazine), 8.90 (s, 1H, C=NH), 9.35 (s, 1H, SO_2_NH); anal. C 50.73, H 3.40, N 9.45% calcd for C_25_H_20_Cl_2_N_4_O_5_S_2_, C 50.76, H 3.41, N 9.47%.

*4-Chloro-2-(2,3-dihydrobenzo[b][1,4]-dioxan-2-ylmethylthio)-5-methyl-N-[imino-(4-methyl-1-oxo-(1H)-phthalazin-2-yl)methyl]benzenesulfonamide* (**23**). Starting from **15** (0.222 g) with stirring for 5 h, 0.126 g (44%) of the title compound **23** was obtained, mp 156–160 °C; IR (KBr) ν_max_ 3331, 3211 (NH), 2923, 2847 (C-H), 1666 (C=O), 1637 (NH), 1523, 1494 (C=C, C=N), 1343, 1145 (SO_2_) cm^−1^; ^1^H-NMR (200 MHz, DMSO-*d_6_*) *δ* 2.20 (s, 3H, CH_3_Ph), 2.47 (s, 3H, CH_3_), 3.46–3.49 (m, 2H, SCH_2_), 4.04–4.10 (m, 1H, OCH), 4.35–4.39 (m, 2H, OCH_2_), 6.71–6.81 (m, 4H, arom.), 7.74 (s, 1H, H-3), 7.78 (s, 1H, H-6), 7.92–8.03 (m, 3H, H-5, H-6, H-7 phthalazine), 8.23–8.27 (d, *J* = 7.8 Hz, 1H, H-8 phthalazine), 8.94 (s, 1H, C=NH), 9.30 (s, 1H, SO_2_NH); anal. C 54.63, H 4.03, N 9.79% calcd for C_26_H_23_ClN_4_O_5_S_2_, C 54.68, H 4.06, N 9.81%.

*4-Chloro-5-methyl-2-(4-methylbenzylthio)-N-[imino-(4-methyl-1-oxo-(1H)-phthalazin-2-yl)methyl]-benzenesulfonamide* (**24**). Starting from **16** (0.200 g) with stirring for 6 h, 0.081 g (30%) of the title compound **24** was obtained, mp 174–180 °C; IR (KBr) ν_max_ 3372, 3231 (NH), 2921, 2853 (C-H), 1685 (C=O), 1634 (NH), 1575, 1456 (C=C, C=N), 1344, 1145 (SO_2_) cm^−1^; ^1^H-NMR (500 MHz, DMSO-*d_6_*) *δ* 2.17 (s, 3H, CH_3_Ph), 2.22 (s, 3H, CH_3_Ph), 2.45 (s, 3H, CH_3_), 4.27 (s, 2H, SCH_2_), 7.04–7.06 (d, *J* = 7.9 Hz, 2H, arom.), 7.29–7.30 (d, *J* = 7.9 Hz, 2H, arom.), 7.47 (s, 1H, H-3), 7.73 (s, 1H, H-6), 7.90–8.02 (m, 3H, H-5, H-6, H-7 phthalazine), 8.26–8.28 (d, *J* = 7.8 Hz, 1H, H-8 phthalazine), 8.89 (s, 1H, C=NH), 9.26 (s, 1H, SO_2_NH); ^13^C-NMR (125 MHz, DMSO-*d_6_*) *δ* 19.1, 19.5, 21.4, 37.1, 126.8, 127.1, 127.6, 128.9, 129.6, 129.8, 129.9, 131.4, 132.7, 133.1, 133.7, 135.1, 137.1, 137.2, 137.9, 138.5, 144.9, 155.0, 157.9; anal. C 56.95, H 4.36, N 10.67% calcd for C_25_H_23_ClN_4_O_3_S_2_, C 56.97, H 4.40, N 10.63%.

*4-Chloro-2-(4-chlorobenzylthio)-5-methyl-N-[imino-(4-methyl-1-oxo-(1H)-phthalazin-2-yl)methyl]-benzenesulfonamide* (**25**). Starting from **17** (0.210 g) with stirring for 1 h, 0.160 g (53%) of the title compound **25** was obtained, mp 169–174 °C; IR (KBr) ν_max_ 3230 (NH), 2920 (C-H), 1683 (C=O), 1634 (NH), 1575, 1487 (C=C, C=N), 1344, 1144 (SO_2_) cm^−1^; ^1^H-NMR (500 MHz, DMSO-*d_6_*) *δ* 2.16 (s, 3H, CH_3_Ph), 2.43 (s, 3H, CH_3_), 4.33 (s, 2H, SCH_2_), 7.29–7.30 (d, *J* = 7.8 Hz, 2H, arom.), 7.43–7.47 (m, 3H, H-3, arom.), 7.72 (s, 1H, H-6), 7.90–8.02 (m, 3H, H-5, H-6, H-7 phthalazine), 8.26–8.28 (d, *J* = 7.8 Hz, 1H, H-8 phthalazine), 8.92 (s, 1H, C=NH), 9.27 (s, 1H, SO_2_NH); ^13^C-NMR (125 MHz, DMSO-*d_6_*) *δ* 19.1, 19.5, 36.5, 126.8, 127.1, 127.5, 129.0, 129.3, 129.9, 131.4, 131.7, 132.5, 133.0, 133.2, 135.1, 136.1, 136.5, 137.9, 138.9, 144.9, 155.0, 157.9; anal. C 52.67, H 3.66, N 10.26% calcd for C_24_H_20_Cl_2_N_4_O_3_S_2_, C 52.65, H 3.68, N 10.23%.

*4-Chloro-5-methyl-2-[(2-phenylsulfonyl)ethylthio]-N-[imino-(4-methyl-1-oxo-(1H)-phthalazin-2-yl)-methyl]benzenesulfonamide* (**26**). Starting from **18** (0.238 g) with stirring for 3.5 h, 0.190 g (64%) of the title compound **25** was obtained, mp 193–195 °C; IR (KBr) ν_max_ 3372, 3231 (NH), 2921, 2853 (C-H), 1685 (C=O), 1634 (NH), 1575, 1456 (C=C, C=N), 1344, 1145 (SO_2_) cm^−1^; ^1^H-NMR (200 MHz, DMSO-*d_6_*) *δ* 2.13 (s, 3H, CH_3_Ph), 2.45 (s, 3H, CH_3_), 3.20–3.27 (m, 2H, CH_2_), 3.55–3.63 (m, 2H, CH_2_), 7.45 (s, 1H, H-3), 7.61–7.81 (m, 4H, arom.), 7.89–8.06 (m, 5H, H-5, H-6 and H-7 phthalazine, arom.), 8.16–8.20 (d, *J* = 7.8 Hz, 1H, H-8 phthalazine), 8.91 (s, 1H, C=NH), 9.25 (s, 1H, SO_2_NH); ^13^C-NMR (50 MHz, DMSO-*d_6_*) *δ* 18.7, 19.6, 26.4, 54.3, 126.4, 126.6, 127.1, 128.0,129.4, 129.9, 130.0, 131.1, 132.7, 133.8, 134.3, 134.4, 134.7, 137.9, 138.6, 139.8, 144.6, 154.6, 157.3; anal. C 50.82, H 3.91, N 9.51% calcd for C_25_H_23_ClN_4_O_5_S_3_, C 50.80, H 3.92, N 9.48%.

#### 3.2.4. General Procedure for the Preparation of *N*-(2-Alkylthio-4-chloro-5-methylbenzenesulfonyl)-2-(2-carboxybenzylidene)hydrazinecarboximidamides **27**–**33**

To a suspension of the appropriate 1-amino-2-(2-alkylthio-4-chloro-5-methylbenzenesulfonyl)guanidine (**11**–**17**) (1.5 mmol) in glacial acetic acid (3 mL) 2-formylbenzoic acid (0.225 g, 1.5 mmol) was added. A reaction mixture was stirred at reflux for 1–11 h, the precipitate was filtered off and dried. In this manner the following compounds were obtained:

*N-(2-Benzylthio-4-chloro-5-methylbenzenesulfonyl)-2-(2-carboxybenzylidene)hydrazinecarboximid-amide* (**27**). Starting from **11** (0.576 g) with stirring for 11 h, 0.348 g (45%) of the title compound **27** was obtained, mp 205–207 °C; IR (KBr) ν_max_ 3456, 3345 (NH), 2923 (C-H), 1674 (C=O), 1626 (NH), 1599, 1485 (C=C, C=N), 1345, 1139 (SO_2_) cm^−1^; ^1^H-NMR (200 MHz, DMSO-*d_6_*) *δ* 2.35 (s, 3H, CH_3_Ph), 4.38 (s, 2H, SCH_2_), 7.19–7.29 (m, 4H, C=NH, arom.), 7.39–7.45 (m, 2H, N=CH, arom.), 7.50–7.62 (m, 3H, arom.), 7.80 (s, 1H, H-3), 7.86–7.88 (m, 1H, arom.), 7.93 (brs, 1H, NHN), 8.26–8.28 (d, *J* = 7.4 Hz, 1H, H-3 carboxybenzylidene), 8.83 (s, 1H, H-6), 11.56 (s, 1H, SO_2_NH), 13.35 (brs, 1H, COOH); anal. C 53.39, H 4.11, N 10.86% calcd for C_23_H_21_ClN_4_O_4_S_2_, C 53.43, H 4.09, N 10.84%.

*2-(2-Carboxybenzylidene)-N-[2-(4-trifluoromethylbenzylthio)-4-chloro-5-methylbenzenesulfonyl]-hydrazinecarboximidamide* (**28**). Starting from **12** (0.627 g) with stirring for 1.5 h, 0.684 g (78%) of the title compound **28** was obtained, mp 203–208 °C; IR (KBr) ν_max_ 3452, 3335 (NH), 2923 (C-H), 1670 (C=O), 1630 (NH), 1565, 1486 (C=C, C=N), 1323, 1140 (SO_2_) cm^−1^; ^1^H-NMR (200 MHz, DMSO-*d_6_*) *δ* 2.33 (s, 3H, CH_3_Ph), 4.46 (s, 2H, SCH_2_), 7.24 (brs, 1H, C=NH),7.48–7.67 (m, 7H, N=CH, arom.), 7.82–7.97 (m, 3H, NHN, arom.), 8.26–8.30 (d, *J* = 7.4 Hz, 1H, H-3 carboxy-benzylidene), 8.87 (s, 1H, H-6), 11.58 (s, 1H, SO_2_NH), 13.35 (brs, 1H, COOH); anal. C 49.30, H 3.48, N 9.57% calcd for C_24_H_20_ClF_3_N_4_O_4_S_2_, C 49.27, H 3.45, N 9.58%.

*2-(2-Carboxybenzylidene)-N-[2-(3-trifluoromethylbenzylthio)-4-chloro-5-methylbeneznesulfonyl]-hydrazinecarboximidamide* (**29**). Starting from **13** (0.627 g) with stirring for 1.5 h, 0.639 g (73%) of the title compound **29** was obtained, mp 187–191 °C; IR (KBr) ν_max_ 3454, 3348 (NH), 2922 (C-H), 1663 (C=O), 1625 (NH), 1569, 1486 (C=C, C=N), 1329, 1136 (SO_2_) cm^−1^; ^1^H-NMR (200 MHz, DMSO-*d_6_*) *δ* 2.32 (s, 3H, CH_3_Ph), 4.47 (s, 2H, SCH_2_), 7.25 (brs, 1H, C=NH),7.47–7.64 (m, 5H, N=CH, arom.), 7.71–7.78 (m, 2H, arom.), 7.86–7.93 (m, 3H, NHN, arom.), 8.24–8.28 (d, *J* = 7.4 Hz, 1H, H-3 carboxybenzylidene), 8.83 (s, 1H, H-6), 11.55 (s, 1H, SO_2_NH), 13.35 (brs, 1H, COOH); anal. C 49.25, H 3.12, N 9.54% calcd for C_24_H_20_ClF_3_N_4_O_4_S_2_, C 49.27, H 3.45, N 9.58%.

*2-(2-Carboxybenzylidene)-N-[2-(6-chlorobenzo[d][1,3]dioxol-5-ylmethylthio)-4-chloro-5-methyl-beneznesulfonyl]hydrazinecarboximidamide* (**30**). Starting from **14** (0.693 g) with stirring for 7 h, 0.804 g (90%) of the title compound **30** was obtained, mp 211–213 °C; IR (KBr) ν_max_ 3449, 3342 (NH), 2922 (C-H), 1672 (C=O), 1626 (NH), 1598, 1487 (C=C, C=N), 1360, 1136 (SO_2_) cm^−1^; ^1^H-NMR (200 MHz, DMSO-*d_6_*) *δ* 2.36 (s, 3H, CH_3_Ph), 4.29 (s, 2H, SCH_2_), 5.98 (s, 2H, OCH_2_O), 7.25 (brs, 1H, C=NH),7.49–7.65 (m, 3H, N=CH, arom.), 7.79 (brs, 1H, NHN), 7.86–7.90 (d, *J* = 7.6 Hz, 1H, arom.), 7.96 (s, 1H, H-3), 8.25–8.29 (d, *J* = 7.4 Hz, 1H, H-3 carboxybenzylidene), 8.82 (s, 1H, H-6), 11.52 (s, 1H, SO_2_NH); anal. C 48.45, H 3.38, N 9.46% calcd for C_24_H_20_Cl_2_N_4_O_6_S_2_, C 48.41, H 3.39, N 9.41%.

*2-(2-Carboxybenzylidene)-N-[2-(2,3-dihydrobenzo[b][1,4]-dioxan-2-ylmethylthio)-4-chloro-5-methyl-beneznesulfonyl]hydrazinecarboximidamide* (**31**). Starting from **15** (0.666 g) with stirring for 1 h, 0.639 g (74%) the title compound **31** was obtained, mp 196–202 °C; IR (KBr) ν_max_ 3457, 3347 (NH), 2922 (C-H), 1666 (C=O), 1625 (NH), 1596, 1444 (C=C, C=N), 1345, 1140 (SO_2_) cm^−1^; ^1^H-NMR (200 MHz, DMSO-*d_6_*) *δ* 2.37 (s, 3H, CH_3_Ph), 3.36–3.42 (m, 2H, SCH_2_), 4.01–4.11 (m, 1H, CHO), 4.34–4.39 (m, 2H, CH_2_O), 6-70-6.80 (m, 4H, arom.), 7.29 (brs, 1H, C=NH),7.48–7.66 (m, 3H, N=CH, arom.), 7.86–7.89 (m, 2H, NHN, arom.), 7.98 (s, 1H, H-3), 8.26–8.30 (d, *J* = 7.4 Hz, 1H, H-3 carboxybenzylidene), 8.83 (s, 1H, H-6), 11.56 (s, 1H, SO_2_NH), 13.10 (brs, 1H, COOH); anal. C 52.19, H 3.98, N 9.76% calcd for C_25_H_23_ClN_4_O_6_S_2_, C 52.22, H 4.03, N 9.74%.

*2-(2-Carboxybenzylidene)-N*-[2-(4-methylbenzylthio)-4-chloro-5-methylbeneznesulfonyl]hydrazine-carboximidamide (**32**). Starting from **16** (0.600 g) with stirring for 11 h, 0.726 g (91%) of the title compound **32** was obtained, mp 204–210 °C; IR (KBr) ν_max_ 3456, 3344 (NH), 2922 (C-H), 1674 (C=O), 1627 (NH), 1598, 1485 (C=C, C=N), 1346, 1141 (SO_2_) cm^−1^; ^1^H-NMR (500 MHz, DMSO-*d_6_*) *δ* 2.15 (s, 3H, CH_3_Ph), 2.30 (s, 3H, CH_3_Ph), 4.25 (s, 2H, SCH_2_), 7.02–7.03 (m, 2H, arom.), 7.21 (s, 1H, C=NH), 7.25–7.27 (m, 2H, arom.), 7.49–7.59 (m, 3H, N=CH, arom.), 7.77 (s, 1H, NHN), 7.85 (m, 1H, arom.), 7.89 (s, 1H, H-3), 8.25–8.26 (d, *J* = 7.4 Hz, 1H, H-3 carboxybenzylidene), 8.83 (s, 1H, H-6), 11.53 (s, 1H, SO_2_NH); ^13^C-NMR (125 MHz, DMSO-*d_6_*) *δ* 19.6, 21.3, 36.6, 127.8, 128.2, 129.6, 129.7, 130.3, 130.8, 131.1, 131.3, 132.4, 132.5, 133.7, 134.6, 136.4, 136.9, 137.2, 140.0, 144.2, 155.7, 168.8; anal. C 54.30, H 4.35, N 10.58% calcd for C_24_H_23_ClN_4_O_4_S_2_, C 54.28, H 4.37, N 10.55%.

*2-(2-Carboxybenzylidene)-N-[2-(4-chlorobenzylthio)-4-chloro-5-methylbeneznesulfonyl]hydrazine-carboximidamide* (**33**). Starting from **17** (0.630 g) with stirring for 3 h, 0.786 g (95%) of the title compound **33** was obtained, mp 184–189 °C; IR (KBr) ν_max_ 3456, 3342 (NH), 2920 (C-H), 1672 (C=O), 1627 (NH), 1563, 1486 (C=C, C=N), 1345, 1141 (SO_2_) cm^−1^; ^1^H-NMR (200 MHz, DMSO-*d_6_*) *δ* 2.33 (s, 3H, CH_3_Ph), 4.36 (s, 2H, SCH_2_), 7.24 (brs, 1H, C=NH), 7.28–7.32 (d, *J* = 8.4 Hz, 2H, arom.), 7.41–7.46 (d, *J* = 8.4 Hz, 2H, arom.), 7.53–7.65 (m, 3H, N=CH, arom.), 7.82–7.93 (m, 3H, NH-N, H-3, arom.), 8.26–8.36 (d, *J* = 7.4 Hz, 1H, H-3 carboxybenzylidene), 8.86 (s, 1H, H-6), 11.57 (s, 1H, SO_2_NH), 13.28 (brs, 1H, COOH); anal. C 50.05, H 3.59, N 10.05% calcd for C_23_H_20_Cl_2_N_4_O_4_S_2_, C 50.09, H 3.66, N 10.16%.

#### 3.2.5. General Procedure for the Preparation of 2-Alkylthio-4-chloro-5-methyl-*N*-[imino-(1-oxo-(1*H*)-phthalazin-2-yl)methyl]benzenesulfonamides **34**–**40**

To a suspension of the appropriate *N*-(2-alkylthio-4-chloro-5-methylbeneznesulfonyl)-2-(2-carboxybenzylidene)hydrazinecarboximidamide (**27**–**33**) (0.5 mmol) in toluene (5 mL) *p*-toluenesulfonic acid (0.026 g, 0.15 mmol) was added. A reaction mixture was stirred at reflux for 1–4.5 h, the precipitate was filtered off, dried and crystallized from ethanol (**34**, **36**) acetonitrile (**35**, **37**, **39**–**40**) or *p-*dioxane (**38**). In this manner the following compounds were obtained:

*2-Benzylthio-4-chloro-5-methyl-N-[imino-(1-oxo-(1H)-phthalazin-2-yl)methyl]benzenesulfonamide* (**34**). Starting from **27** (0.258 g) with stirring for 3 h, 0.160 g (64%) of the title compound **34** was obtained, mp 166–170 °C; IR (KBr) ν_max_ 3314, 3199 (NH), 2920 (C-H), 1670 (C=O), 1642 (NH), 1316, 1147 (SO_2_) cm^−1^; ^1^H-NMR (500 MHz, DMSO-*d_6_*) *δ* 2.19 (s, 3H, CH_3_Ph), 4.32 (s, 2H, SCH_2_), 7.22–7.28 (m, 3H, arom.), 7.42–7.43 (m, 2H, arom.), 7.47 (s, 1H, H-3), 7.79 (s, 1H, H-4 phthalazine), 7.91–7.99 (m, 3H, H-5, H-6, H-7 phthalazine), 8.24–8.25 (d, *J* = 7.8 Hz , 1H, H-8 phthalazine), 8.48 (s, 1H, H-6), 8.89 (s, 1H, C=NH), 9.33 (s, 1H, SO_2_NH); ^13^C-NMR (125 MHz, DMSO-*d_6_*) *δ b*19.5, 37.3, 126.7, 127.8, 127.9, 128.1, 129.0, 129.1, 129.8, 129.9, 131.5, 132.8, 133.5, 135.3, 136.8, 137.1, 138.0, 138.3, 139.5, 155.0, 158.0; anal. 55.39, H 3.82, N 11.20% calcd for C_23_H_19_ClN_4_O_3_S_2_, C 55.36, H 3.84, N 11.23%.

*4-Chloro-5-methyl-2-(4-trifluoromethylbenzylthio)-N-[imino-(1-oxo-(1H)-phthalazin-2-yl)methyl]-benzenesulfonamide* (**35**). Starting from **28** (0.293 g) with stirring for 2 h, 0.122 g (43%) of the title compound **35** was obtained, mp 162–167 °C; IR (KBr) ν_max_ 3372 (NH), 2922 (C-H), 1677 (C=O), 1629 (NH), 1554, 1453 (C=C, C=N), 1323, 1141 (SO_2_) cm^−1^; ^1^H-NMR (500 MHz, DMSO-*d_6_*) *δ* 2.19 (s, 3H, CH_3_Ph), 4.44 (s, 2H, SCH_2_), 7.49 (s, 1H, H-3), 7.58–7.60 (d, *J* = 8.0 Hz, 2H, arom.), 7.64–7.65 (d, *J* = 8.0 Hz, 2H, arom.), 7.80 (s, 1H, H-4 phthalazine), 7.91–8.00 (m, 3H, H-5, H-6, H-7 phthalazine), 8.23–8.25 (d, *J* = 7.8 Hz, 1H, H-8 phthalazine), 8.49 (s, 1H, H-6), 8.90 (s, 1H, C=NH), 9.35 (s, 1H, SO_2_NH); ^13^C-NMR (125 MHz, DMSO-*d_6_*) *δ* 19.5, 36.6, 125.8, 125.9, 126.7, 127.8, 128.1, 129.4, 129.9, 130.6, 131.5, 133.3, 133.5, 135.3, 136.1, 138.1, 138.7, 139.6, 142.2, 155.1, 158.0; anal. C 50.80, H 3.15, N 9.85% calcd for C_24_H_18_ClF_3_N_4_O_3_S_2_, C 50.84, H 3.20, N 9.88%.

*4-Chloro-5-methyl-2-(3-trifluoromethylbenzylthio)-N-[imino-(1-oxo-(1H)-phthalazin-2-yl)methyl]-benzenesulfonamide* (**36**). Starting from **29** (0.293 g) with stirring for 1 h, 0.057 g (20%) of the title compound **36** was obtained, mp 158–163 °C; IR (KBr) ν_max_ 3389 (NH), 2923 (C-H), 1672 (C=O), 1640 (NH), 1520, 1451 (C=C, C=N), 1330, 1116 (SO_2_) cm^−1^; ^1^H-NMR (200 MHz, DMSO-*d_6_*) *δ* 2.22 (s, 3H, CH_3_Ph), 4.47 (s, 2H, SCH_2_), 7.50 (s, 1H, H-3), 7.55–7.62 (m, 2H, arom.), 7.74–7.78 (m, 2H, arom.), 7.82 (s, 1H, H-4 phthalazine), 7.89–8.01 (m, 3H, H-5, H-6, H-7 phthalazine), 8.24–8.28 (d, *J* = 7.8 Hz, 1H, H-8 phthalazine), 8.48 (s, 1H, H-6), 8.90 (s, 1H, C=NH), 9.35 (s, 1H, SO_2_NH); anal. C 50.87, H 3.24, N 9.89% calcd for C_24_H_18_ClF_3_N_4_O_3_S_2_, C 50.84, H 3.20, N 9.88%.

*4-Chloro-2-(6-chlorobenzo[d][1,3]dioxol-5-ylmethylthio)-5-methyl-N-[imino-(1-oxo-(1H)-phthalazin-2-yl)methyl]benzenesulfonamide* (**37**). Starting from **30** (0.298 g) with stirring for 2 h, 0.101 g (35%) of the title compound **37** was obtained, mp 180–185 °C; IR (KBr) ν_max_ 3363, 3236 (NH), 2923, 2853 (C-H), 1676 (C=O), 1633 (NH), 1532, 1480 (C=C, C=N), 1344, 1138 (SO_2_) cm^−1^; ^1^H-NMR (200 MHz, DMSO-*d_6_*) *δ* 2.25 (s, 3H, CH_3_Ph), 4.31 (s, 2H, SCH_2_), 6.02 (s, 2H, OCH_2_O), 7.07 (s, 1H, arom.), 7.12 (s, 1H, arom.), 7.45 (s, 1H, H-3), 7.85 (s, 1H, H-4 phthalazine), 7.94–8.04 (m, 3H, H-5, H-6, H-7 phthalazine), 8.25–8.28 (d, *J* = 7.8 Hz, 1H, H-8 phthalazine), 8.48 (s, 1H, H-6), 8.90 (s, 1H, C=NH), 9.35 (s, 1H, SO_2_NH); anal. C 49.90, H 3.15, N 9.68% calcd for C_25_H_20_Cl_2_N_4_O_5_S_2_, C 49.92, H 3.14, N 9.70%.

*4-Chloro-2-(2,3-dihydrobenzo[b][1,4]-dioxan-2-ylmethylthio)-5-methyl-N-[imino-(1-oxo-(1H)-phthalazin-2-yl)methyl]benzenesulfonamide* (**38**). Starting from **31** (0.288 g) with stirring for 2 h, 0.175 g (63%) of the title compound **38** was obtained, mp 195–199 °C; IR (KBr) ν_max_ 3380 (NH), 2920 (C-H), 1669 (C=O), 1636 (NH), 1526, 1491 (C=C, C=N), 1344, 1135 (SO_2_) cm^−1^; ^1^H-NMR (200 MHz, DMSO-*d_6_*) *δ* 2.23 (s, 3H, CH_3_Ph), 3.46–3.49 (m, 2H, SCH_2_), 4.05–4.10 (m, 1H, OCH), 4.35–4.40 (m, 2H, OCH_2_), 6.73–6.81 (m, 4H, arom.), 7.74 (s, 1H, H-3), 7.84 (s, 1H, H-4 phthalazine), 7.89–8.02 (m, 3H, H-5, H-6, H-7 phthalazine), 8.20–8.24 (d, *J* = 7.8 Hz, 1H, H-8 phthalazine), 8.45 (s, 1H, H-6), 8.94 (s, 1H, C=NH), 9.39 (s, 1H, SO_2_NH); anal. C 53.87, H 3.81, N 10.03% calcd for C_25_H_21_ClN_4_O_5_S_2_, C 53.90, H 3.80, N 10.06%.

*4-Chloro-5-methyl-2-(4-methylbenzylthio)-N-[imino-(1-oxo-(1H)-phthalazin-2-yl)methyl]benzene-sulfonamide* (**39**). Starting from **32** (0.114 g) with stirring for 4.5 h, 0.164 g (64%) of the title compound **39** was obtained, mp 184–186 °C; IR (KBr) ν_max_ 3364, 3227 (NH), 2920, 2854 (C-H), 1681 (C=O), 1630 (NH), 1556, 1453 (C=C, C=N), 1339, 114 (SO_2_) cm^−1^; ^1^H-NMR (200 MHz, DMSO-*d_6_*) *δ* 2.24 (s, 3H, CH_3_Ph), 2.26 (s, 3H, CH_3_Ph), 4.31 (s, 2H, SCH_2_), 7.06–7.11 (d, *J* = 8.0 Hz, 2H, arom.), 7.31–7.35 (d, *J* = 8.0 Hz, 2H, arom.), 7.51 (s, 1H, H-3), 7.82 (s, 1H, H-4 phthalazine), 7.91–8.05 (m, 3H, H-5, H-6, H-7 phthalazine), 8.26–8.30 (d, *J* = 7.8 Hz, 1H, H-8 phthalazine), 8.52 (s, 1H, H-6), 8.90 (s, 1H, C=NH), 9.35 (s, 1H, SO_2_NH); anal. C 56.25, H 4.12, N 11.00% calcd for C_24_H_21_ClN_4_O_3_S_2_, C 56.19, H 4.13, N 10.92%.

*4-Chloro-2-(4-chlorobenzylthio)-5-methyl-N-[imino-(1-oxo-(1H)-phthalazin-2-yl)methyl]benzene-sulfonamide* (**40**). Starting from **33** (0.276 g) with stirring for 1.5 h, 0.100 g (37%) of the title compound **40** was obtained, mp 164–168 °C; IR (KBr) ν_max_ 3363, 3229 (NH), 2921 (C-H), 1679 (C=O), 1630 (NH), 1557, 1489 (C=C, C=N), 1337, 1142 (SO_2_) cm^−1^; ^1^H-NMR (500 MHz, DMSO-*d_6_*) *δ* 2.20 (s, 3H, CH_3_Ph), 4.34 (s, 2H, SCH_2_), 7.30–7.31 (d, *J* = 8.0 Hz, 2H, arom.), 7.44–7.47 (m, 3H, H-3, arom.), 7.79 (s, 1H, H-4 phthalazine), 7.92–8.00 (m, 3H, H-5, H-6, H-7 phthalazine), 8.24–8.25 (d, *J* = 7.8 Hz, 1H, H-8 phthalazine), 8.49 (s, 1H, H-6), 8.89 (s, 1H, C=NH), 9.33 (s, 1H, SO_2_NH); ^13^C-NMR (125 MHz, DMSO-*d_6_*) *δ b*19.5, 36.4, 126.7, 127.8, 128.1, 129.0, 129.3, 129.9, 131.5, 131.6, 132.5, 133.1, 133.5, 135.3, 136.1, 136.5, 138.0, 138.6, 139.6, 155.1, 158.0; anal. C 51.77, H 3.36, N 10.50% calcd for C_23_H_18_Cl_2_N_4_O_3_S_2_, C 51.78, H 3.40, N 10.50%.

### 3.3. Antifungal Activity

The study involved 27 of patients in the 37–85 years old age range, with oral and oropharyngeal candidosis (11 patients), neoplastic disease (two patients), diabetes mellitus (three patients), patients after chemotherapy and radiotherapy (three patients), antibiotic (four patients) and steroid (two patients) therapy, wearing a dental prosthesis—18 patients). One (23 materials) or two (four materials) isolates per patient were included in this study. The strains were identified by standard morphological and biochemical methods (API tests-system, bioMerieux, Durham, NC, USA) [34,35]. A total of 31 strains belonging to the genera of *Candida* (26 strains), *Geotrichum* (two), *Rhodotorula* (two) and *Saccharomyces* (one) isolated from the patients were used for testing. The susceptibility (MIC) of fungi was determined by means of the dilution technique in the agar. The compounds were dissolved in 1 mL of dimethylsulfoxide (DMSO) immediately before the experiments. Further dilutions were performed using sterile distilled water. The following concentrations of compounds were used: 200, 100, 50, 25, 12.5 and 6.2 µg/mL. Fluconazole (Fluka, Buchs, Switzerland) was applied as a reference antifungal agent. Stock solutions were prepared by dissolving in DMSO. Final concentrations of fluconazole ranging from 3.1 to 100 µg/mL were used. Adequate concentrations of each compound and fluconazole were added to Sabouraud’s agar. The agar plate without compounds were the control growth of the fungal strains.

Inocula containing 10^5^ colony forming units (CFU) per spot was seeded with a Steers replicator applied on the surface of the agar. The inoculated compound and compound-free agar plates were incubated under aerobic conditions for 24 h at 37 °C. The MIC was defined as the lowest concentration of the compound that completely inhibited the growth of yeast-like fungi. For some strains (*Geotrichum candidum* , *Rhodotorula mucilaginosa* and *Saccharomyces cerevisiae*) incubation was prolonged up to 48 h before carrying out the first reading.

## 4. Conclusions

We have developed facile methods for the synthesis of 2-alkylthio-4-chloro-5-methyl-*N*-[imino-(1-oxo-(1*H*)-phthalazin-2-yl)methyl]benzenesulfonamide derivatives **19**–**26**, **34**–**40** by the reactions of the appropriate aminoguanidines **11**–**18** with *ortho-*carbonyl substituted benzoic acids. Fifteen of the new *N*-[imino-(1-oxo-(1*H*)-phthalazin-2-yl)methyl]benzenesulfonamide derivatives were screened *in vitro* for their antifungal effects against yeast-like fungi isolated from the oral cavity and respiratory tract of patients with candidiasis. Many of them have shown significant growth-inhibitory activity, superior or comparable to those of reference drug fluconazole. Regarding the structure-activity relationships, we conclude that in general the lack of a methyl group at position 4 of the 1-phthalazinone system enhanced the antifungal activity. Interestingly, compounds **22**–**24**, **26**, **34**, **36**, **37** and **40** exhibited the most prominent activities against *Candida albicans*, inhibiting at least 1–3 (12.5%–37.5%) of the eight tested strains at the low MIC level of ≤6.2–25 µg/mL, thus they may be the promising leads for further development as antifungal agents in the treatment of candidiasis.

## Supplementary Materials

Supplementary materials can be accessed at: http://www.mdpi.com/1420-3049/19/9/13704/s1.

## Supplementary Files

Supplementary File 1
