# Supplementary material for: Synthesis and Anti-Yeast Evaluation of Novel 2-Alkylthio-4-chloro-5-methyl-N-[imino-(1-oxo-(1H)-phthalazin-2-yl)methyl]benzenesulfonamide Derivatives
Source: Molecules. 2014 Sep 2;19(9):13704–23. doi: 10.3390/molecules190913704 (PMC6271044; doi:10.3390/molecules190913704)

Supplementary Materials

Table of Contents

Table S1. Detailed antifungal activity of compounds **19–26** and **34–40**. S2
Spectrum 1. ^{1}H-NMR of compound **21** (500 MHz, DMSO-d_6). S5
Spectrum 2. ^{13}C-NMR of compound **21** (125 MHz, DMSO-d_6). S6
Spectrum 3. ^{1}H-NMR of compound **24** (500 MHz, DMSO-d_6). S7
Spectrum 4. ^{13}C-NMR of compound **24** (125 MHz, DMSO-d_6). S8
Spectrum 5. ^{1}H-NMR of compound **25** (500 MHz, DMSO-d_6). S9
Spectrum 6. ^{13}C-NMR of compound **25** (125 MHz, DMSO-d_6). S10
Spectrum 7. ^{1}H-NMR of compound **32** (500 MHz, DMSO-d_6). S11
Spectrum 8. ^{13}C-NMR of compound **32** (125 MHz, DMSO-d_6). S12
Spectrum 9. ^{1}H-NMR of compound **34** (500 MHz, DMSO-d_6). S13
Spectrum 10. ^{13}C-NMR of compound **34** (125 MHz, DMSO-d_6). S14
Spectrum 11. ^{1}H-NMR of compound **35** (500 MHz, DMSO-d_6). S15
Spectrum 12. ^{13}C-NMR of compound **35** (125 MHz, DMSO-d_6). S16
Spectrum 13. ^{1}H-NMR of compound **40** (500 MHz, DMSO-d_6). S17
Spectrum 14. ^{13}C-NMR of compound **40** (125 MHz, DMSO-d_6). S18

Table S1. Detail antifungal activity of compounds **19**–**26** and **34**–**40**.

**Compound**	**MIC [µg/mL]**	**STRAIN / NUMBER OF STRAINS**										
		Candida albicans	*Candida glabrata*	*Candida guilliermondii*	*Candida krusei*	*Candida lusitaniae*	*Candida parapsilosis*	*Candida tropicalis*	*Candida utilis*	*Geotrichum candidum*	*Rhodothorula mucilaginosa*	*Saccharomyces cerevisiae*
		8	4	2	3	2	3	3	1	2	2	1
**19**	**≥200**	4	4	2	3	1	3	3		2		1
	**100**	2				1			1			
	**50**	2									1	
	**25**										1	
	**12.5**											
	**≤6.2**											
**20**	**≥200**	2	4		3	2	2	2		1	2	1
	**100**	6		1				1	1	1		
	**50**						1					
	**25**			1								
	**12.5**											
	**≤6.2**											
**21**	**≥200**	2	4		3	1	2	2		2	2	1
	**100**	6		1		1						
	**50**			1			1	1	1			
	**25**											
	**12.5**											
	**≤6.2**											
**22**	**≥200**	5	4		3	1	2	2	1	2	2	1
	**100**	1		1		1	1	1				
	**50**			1								
	**25**	1										
	**12.5**	1										
	**≤6.2**											
**23**	**≥200**	4	4		3	2	2	2	1	2	2	1
	**100**	1		1								
	**50**						1	1				
	**25**	3		1								
	**12.5**											
	**≤6.2**											
**24**	**≥200**	4	3	2	2	2	3	3	1	2		1
	**100**		1									
	**50**	1			1							
	**25**	3										
	**12.5**										1	
	**≤6.2**										1	
**25**	**≥200**	2	4		3	2	1	2				1
	**100**	3		1						2	2	
	**50**	3					1		1			
	**25**											
	**12.5**			1			1	1				
	**≤6.2**											

Table S1. *Cont.*

Compound	MIC [µg/mL]	STRAIN NUMBER OF STRAINS										
		Candida albicans	*Candida glabrata*	*Candida guilliermondii*	*Candida krusei*	*Candida lusitaniae*	*Candida parapsilosis*	*Candida tropicalis*	*Candida utilis*	*Geotrichum candidum*	*Rhodothorula mucilaginosa*	*Saccharomyces cerevisiae*
		8	4	2	3	2	3	3	1	2	2	1
**26**	**≥200**	4	1	2	1	3	3		2		1	1
	**100**	3	1	1	1			1				
	**50**											
	**25**	1								1		
	**12.5**											
	**≤6.2**											
**34**	**≥200**	1	4		2		2	2				
	**100**	4			1						1	1
	**50**	1				1		1		1	1	
	**25**	2		2		1			1			
	**12.5**									1		
	**≤6.2**						1					
**35**	**≥200**	4	4	1	3	1	2	2		2	2	1
	**100**	1				1	1					
	**50**	3		1				1	1			
	**25**											
	**12.5**											
	**≤6.2**											
**36**	**≥200**	3	4		3		2	2		2	2	1
	**100**	3		1		1			1			
	**50**						1	1				
	**25**					1						
	**12.5**	1		1								
	**≤6.2**	1										
**37**	**≥200**	3	4	2	3	2	3	3		2		1
	**100**	1										
	**50**	2							1		1	
	**25**	1									1	
	**12.5**	1										
	**≤6.2**											
**38**	**≥200**	5	2		2	1	2	2		2	1	1
	**100**	3	2	1		1		1	1		1	
	**50**			1	1							
	**25**											
	**12.5**											
	**≤6.2**						1					

Table S1. *Cont.*

Compound	MIC [µg/mL]	STRAIN NUMBER OF STRAINS										
		Candida albicans	*Candida glabrata*	*Candida guilliermondii*	*Candida krusei*	*Candida lusitaniae*	*Candida parapsilosis*	*Candida tropicalis*	*Candida utilis*	*Geotrichum candidum*	*Rhodothorula mucilaginosa*	*Saccharomyces cerevisiae*
		8	4	2	3	2	3	3	1	2	2	1
**39**	**≥200**	6	4		3		3	3	1	2		1
	**100**			2		2						
	**50**	2									1	
	**25**										1	
	**12.5**											
	**≤6.2**											
**40**	**≥200**	1	2		3		2	2			2	
	**100**	3	2	1		1				2		1
	**50**	3				1	1	1				
	**25**	1		1								
	**12.5**											
	**≤6.2**								1			
**Fluconazole**	**≥100**	6	2		(1)			1(3)		(1)	2	1
	**50**	1	1		2(1)			1		(1)		
	**25**	(1)	1	1	1	(1)	1	1	1	1		
	**12.5**	1		1		2(1)	1			1		
	**6.2**						1					
	**≤3.1**											

Spectrum 1. ^{1}H-NMR of compound **21** (500 MHz, DMSO-d_6).

No.	(ppm)	(Hz)	Height
1	2.16	1079.4	0.1769
2	2.42	1207.3	0.2103
3	4.44	2221.5	0.1349
4	7.47	3735.3	0.0855
5	7.49	3742.6	0.0456
6	7.50	3750.4	0.0286
7	7.57	3784.6	0.0316
8	7.59	3791.9	0.0275
9	7.74	3868.6	0.0680
10	7.75	3875.4	0.0732
11	7.89	3944.8	0.0155
12	7.91	3952.1	0.0347
13	7.92	3959.4	0.0273
14	7.95	3973.1	0.0246
15	7.96	3980.4	0.0493
16	7.98	3990.7	0.0332
17	8.00	3998.0	0.0349
18	8.01	4004.8	0.0165
19	8.25	4124.5	0.0297
20	8.27	4132.3	0.0313
21	8.93	4460.9	0.0412
22	9.28	4639.6	0.0445

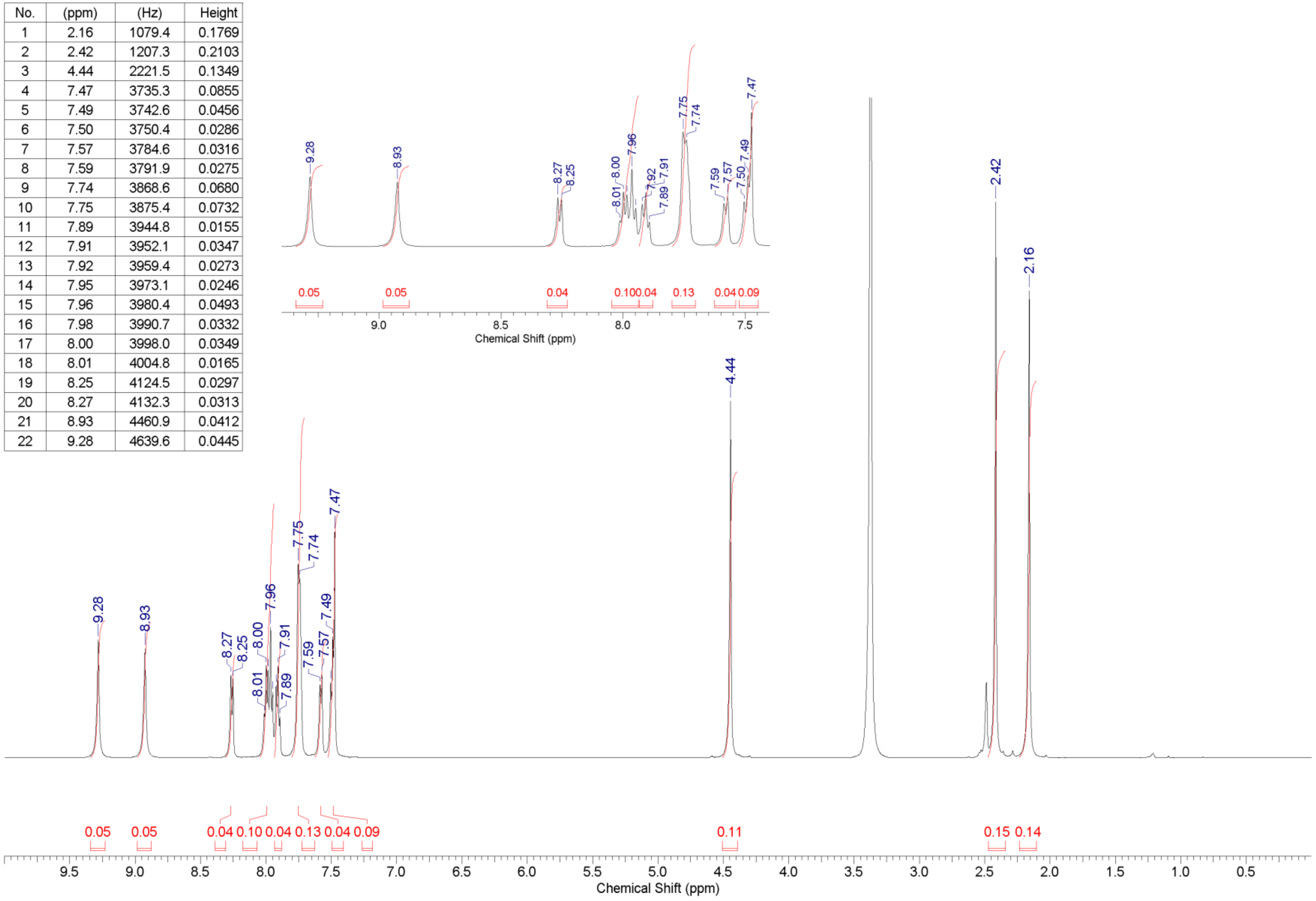

Spectrum 2. ^{13}C-NMR of compound **21** (125 MHz, DMSO-d_6).

No.	(ppm)	(Hz)	Height
1	19.04	2393.7	0.2200
2	19.53	2455.1	0.1852
3	36.72	4615.1	0.1192
4	124.65	15667.9	0.0414
5	124.68	15671.4	0.0632
6	126.48	15897.6	0.0685
7	126.81	15938.4	0.1240
8	127.06	15970.9	0.1264
9	127.55	16031.9	0.0912
10	129.69	16300.7	0.1110
11	129.84	16320.4	0.0355
12	129.88	16324.4	0.1124
13	130.18	16362.1	0.1684
14	131.48	16525.9	0.1316
15	133.13	16732.7	0.1402
16	133.29	16753.8	0.0989
17	133.97	16838.6	0.1334
18	135.14	16985.7	0.0970
19	136.02	17096.3	0.1027
20	137.90	17333.4	0.1020
21	138.74	17438.4	0.1208
22	139.11	17484.9	0.0890
23	144.93	18216.1	0.0905
24	155.11	19496.1	0.0825
25	157.88	19843.8	0.0952

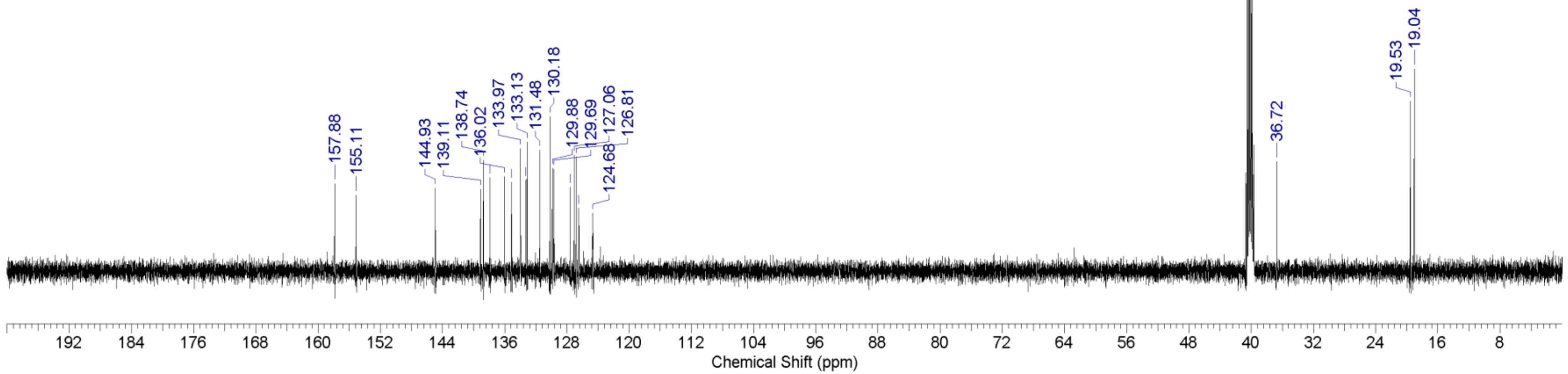

Spectrum 3. ^{1}H-NMR of compound **24** (500 MHz, DMSO-d_6).

No.	(ppm)	(Hz)	Height
1	2.17	1083.3	0.1054
2	2.22	1110.6	0.1147
3	2.45	1223.4	0.1337
4	4.27	2134.6	0.0778
5	7.04	3519.9	0.0312
6	7.06	3527.8	0.0367
7	7.29	3642.5	0.0388
8	7.30	3650.3	0.0359
9	7.47	3733.8	0.0456
10	7.73	3864.7	0.0247
11	7.90	3950.1	0.0084
12	7.92	3957.0	0.0184
13	7.93	3964.8	0.0127
14	7.96	3978.0	0.0126
15	7.97	3985.8	0.0266
16	7.99	3995.1	0.0169
17	8.01	4002.4	0.0170
18	8.02	4009.7	0.0062
19	8.26	4130.3	0.0171
20	8.28	4138.1	0.0162
21	8.89	4445.3	0.0202
22	9.26	4629.9	0.0217

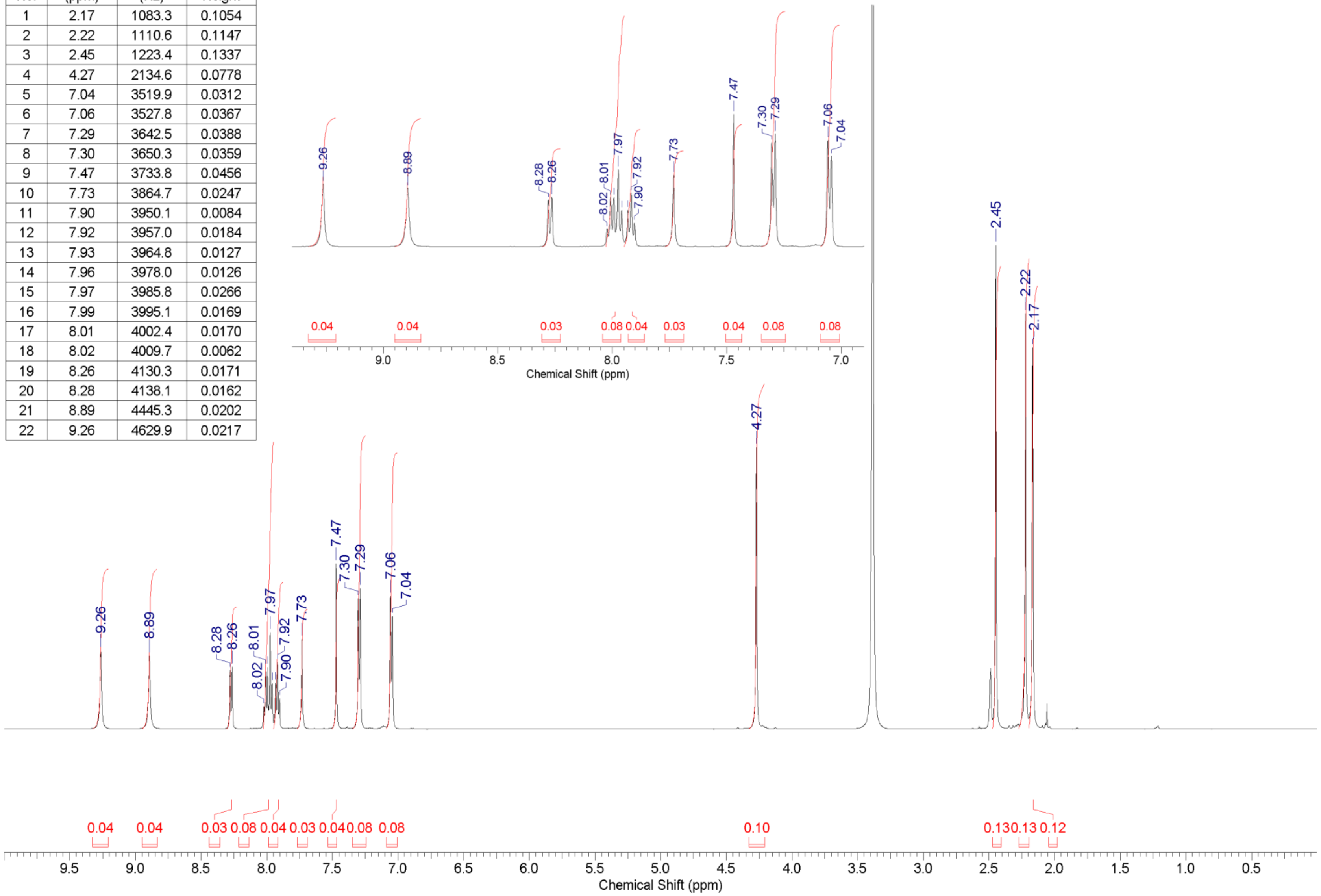

Spectrum 4. ^{13}C-NMR of compound **24** (125 MHz, DMSO-d_6).

No.	(ppm)	(Hz)	Height
1	19.11	2402.0	0.2416
2	19.53	2454.7	0.2015
3	21.38	2687.4	0.2036
4	37.09	4662.1	0.1547
5	126.81	15939.3	0.1668
6	127.10	15974.8	0.1690
7	127.57	16035.0	0.1107
8	128.88	16198.8	0.1298
9	129.64	16294.5	0.4877
10	129.77	16310.8	0.4868
11	129.89	16325.7	0.1268
12	131.40	16515.8	0.1491
13	132.67	16675.7	0.1284
14	133.14	16734.1	0.1596
15	133.69	16803.4	0.1571
16	135.14	16986.1	0.1335
17	137.08	17229.8	0.1491
18	137.24	17250.0	0.1257
19	137.93	17336.1	0.1188
20	138.54	17413.8	0.1069
21	144.92	18215.2	0.1049
22	155.02	19485.1	0.0984
23	157.89	19846.0	0.1115

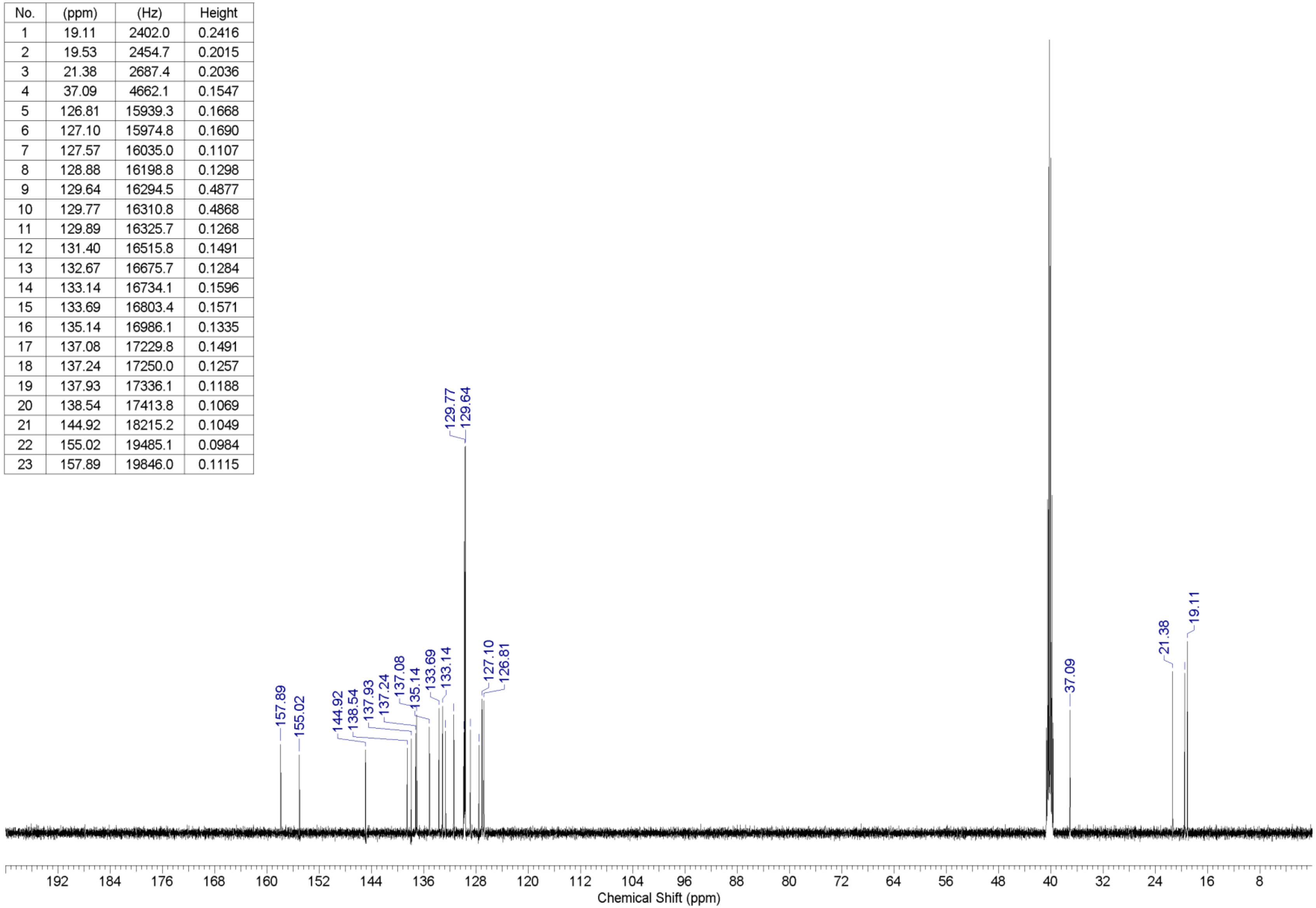

Spectrum 5. ^{1}H-NMR of compound **25** (500 MHz, DMSO-d_6).

No.	(ppm)	(Hz)	Height
1	2.16	1079.4	0.1320
2	2.43	1215.1	0.1587
3	4.33	2166.3	0.1026
4	7.29	3642.0	0.0361
5	7.30	3649.8	0.0505
6	7.43	3714.8	0.0503
7	7.45	3723.1	0.0487
8	7.47	3733.8	0.0593
9	7.72	3859.8	0.0309
10	7.90	3949.7	0.0109
11	7.92	3957.0	0.0248
12	7.93	3964.3	0.0186
13	7.96	3977.5	0.0171
14	7.97	3985.3	0.0356
15	7.99	3994.6	0.0239
16	8.01	4001.9	0.0239
17	8.02	4009.2	0.0103
18	8.26	4128.4	0.0218
19	8.28	4136.2	0.0220
20	8.92	4457.5	0.0288
21	9.27	4633.8	0.0315

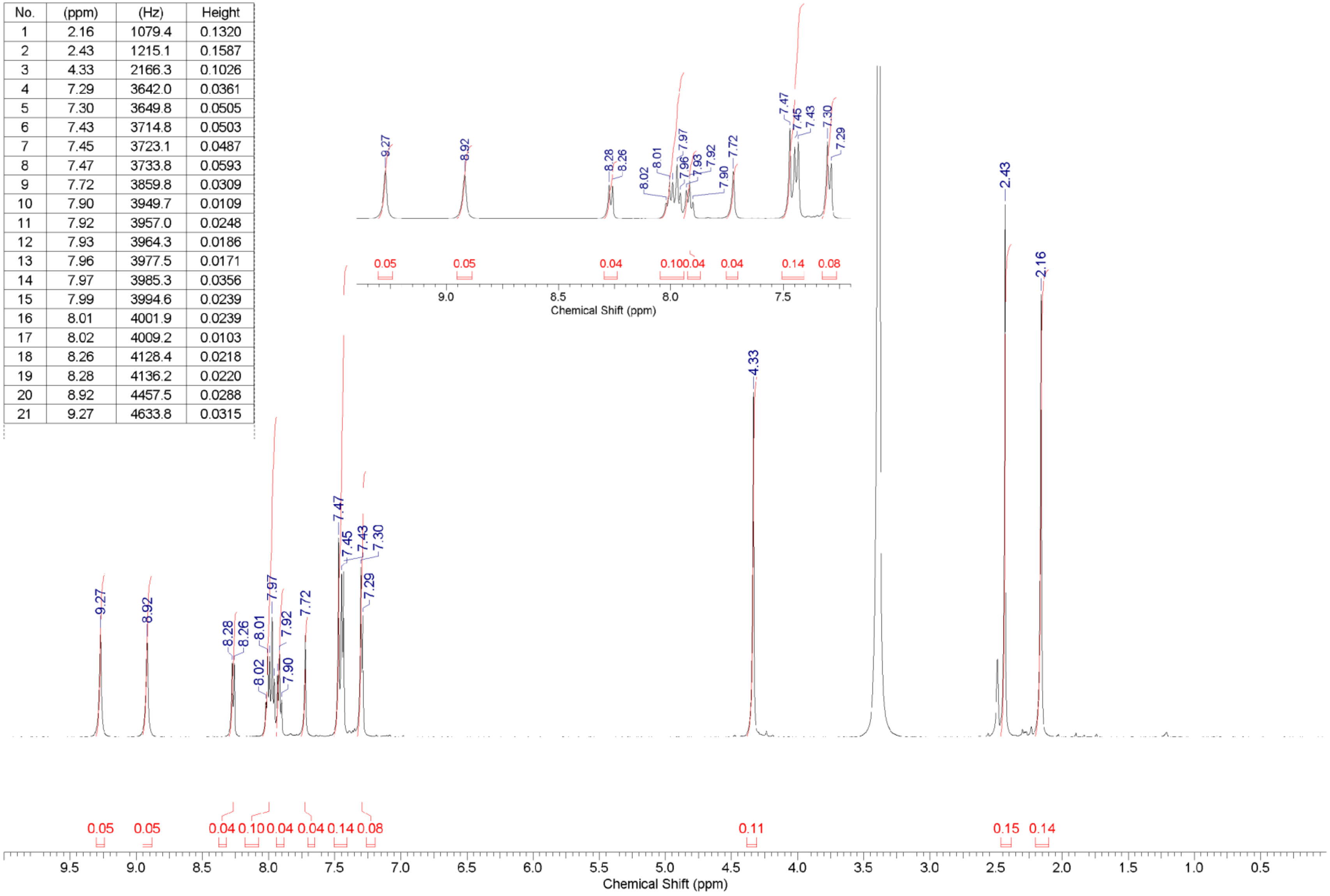

Spectrum 6. ^{13}C-NMR of compound **25** (125 MHz, DMSO-d_6).

No.	(ppm)	(Hz)	Height
1	19.09	2399.4	0.1757
2	19.54	2456.5	0.1455
3	36.49	4586.6	0.0961
4	126.84	15942.4	0.1131
5	127.08	15973.1	0.1081
6	127.55	16031.9	0.0839
7	129.01	16215.9	0.3762
8	129.26	16246.7	0.0834
9	129.88	16324.8	0.0864
10	131.44	16520.7	0.1020
11	131.67	16549.6	0.3546
12	132.48	16651.5	0.0960
13	133.03	16720.9	0.0807
14	133.16	16737.1	0.1078
15	135.15	16987.4	0.0832
16	136.14	17111.7	0.1055
17	136.49	17155.6	0.0904
18	137.93	17336.5	0.0849
19	138.87	17455.1	0.0740
20	144.93	18216.9	0.0833
21	155.04	19487.3	0.0708
22	157.87	19843.4	0.0839

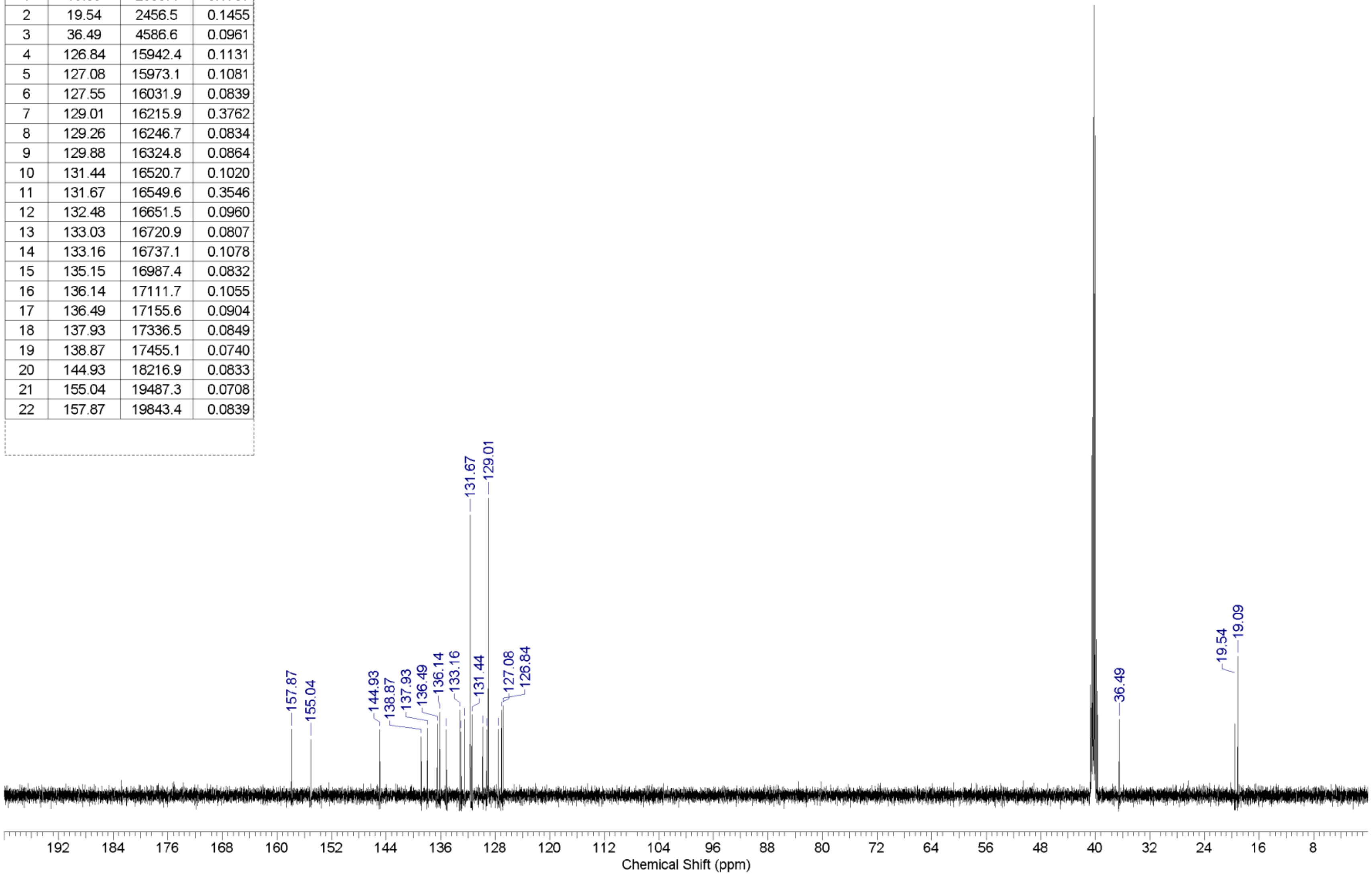

Spectrum 7. ^{1}H-NMR of compound **32** (500 MHz, DMSO-d_6).

No.	(ppm)	(Hz)	Height
1	2.15	1076.4	1.0000
2	2.16	1079.4	0.9535
3	2.30	1148.7	0.9914
4	2.30	1151.6	0.9414
5	4.25	2125.8	0.7267
6	7.02	3509.2	0.4026
7	7.03	3513.1	0.3836
8	7.21	3602.5	0.1727
9	7.25	3625.4	0.3910
10	7.26	3628.4	0.4526
11	7.27	3632.7	0.3762
12	7.49	3745.5	0.5446
13	7.50	3748.0	0.5200
14	7.58	3786.6	0.1504
15	7.58	3790.5	0.1742
16	7.59	3792.9	0.1759
17	7.77	3884.2	0.1680
18	7.85	3921.8	0.1683
19	7.85	3924.8	0.1942
20	7.86	3928.7	0.1644
21	7.87	3931.6	0.1489
22	7.89	3945.8	0.2946
23	7.90	3948.7	0.2751
24	8.25	4121.1	0.1652
25	8.25	4124.0	0.1886
26	8.26	4128.4	0.1663
27	8.83	4412.6	0.3418
28	8.83	4415.5	0.3243
29	11.53	5762.8	0.3322

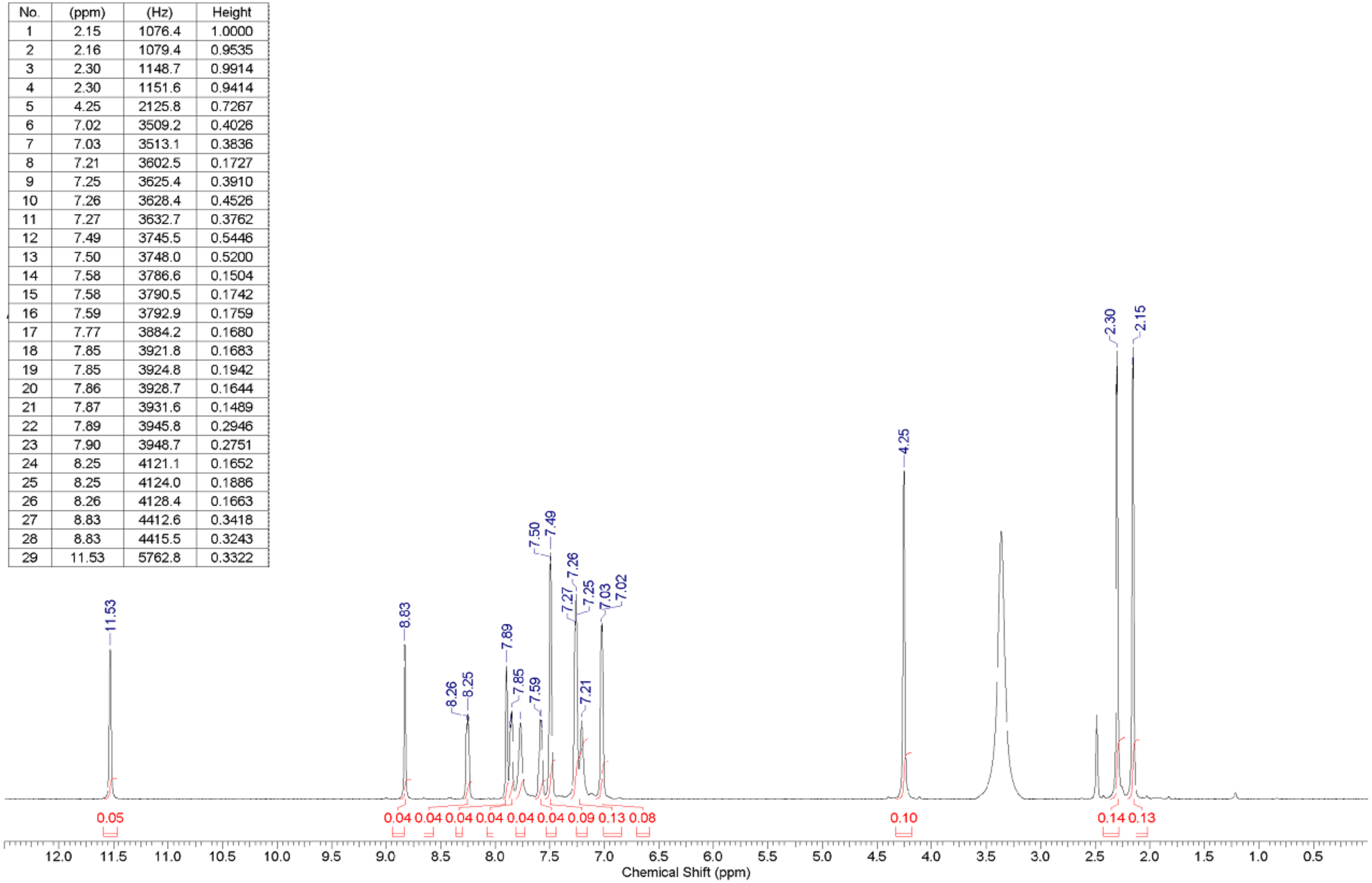

Spectrum 8. ^{13}C-NMR of compound **32** (125 MHz, DMSO-d_6).

No.	(ppm)	(Hz)	Height
1	19.62	2466.1	0.1341
2	21.31	2678.2	0.1182
3	36.59	4598.9	0.0917
4	127.78	16060.5	0.0774
5	128.22	16116.7	0.0821
6	129.65	16295.8	0.3352
7	129.69	16301.5	0.3050
8	130.26	16372.2	0.0716
9	130.81	16442.1	0.0846
10	131.15	16484.7	0.0958
11	131.27	16499.6	0.0693
12	132.40	16641.4	0.0947
13	132.46	16649.3	0.0923
14	133.72	16807.4	0.0854
15	134.62	16920.2	0.0751
16	136.41	17145.1	0.0750
17	136.99	17218.0	0.0726
18	137.23	17248.3	0.0721
19	140.04	17601.3	0.0753
20	144.17	18120.8	0.0779
21	155.67	19565.9	0.0849
22	168.79	21215.2	0.0732

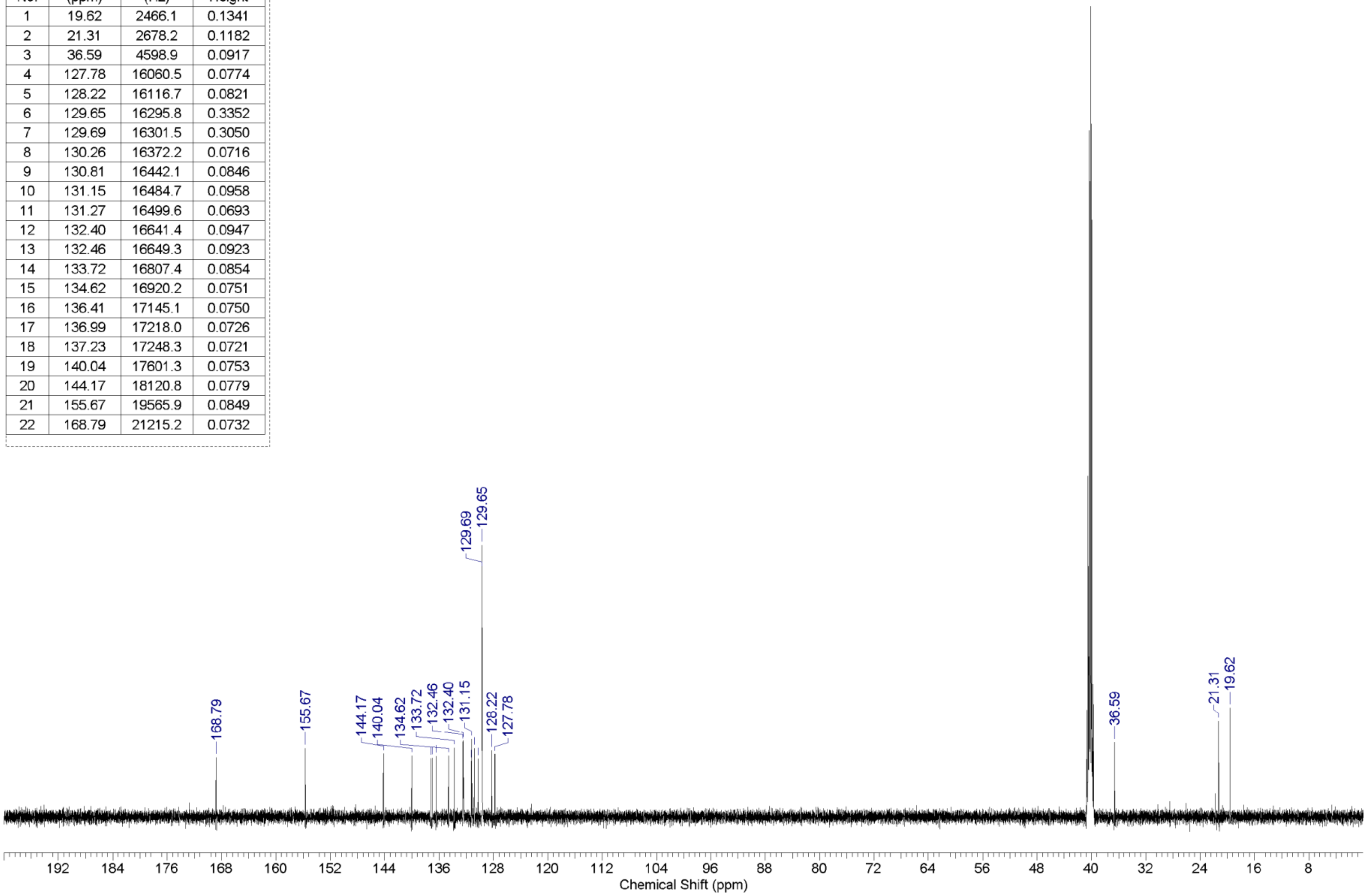

Spectrum 9. ^{1}H-NMR of compound **34** (500 MHz, DMSO-d_6).

No.	(ppm)	(Hz)	Height
1	2.19	1096.9	0.4180
2	4.32	2161.0	0.3401
3	7.22	3606.4	0.0670
4	7.23	3613.7	0.0765
5	7.25	3624.4	0.1113
6	7.27	3631.8	0.1691
7	7.28	3639.1	0.1030
8	7.42	3707.0	0.1541
9	7.43	3714.3	0.1560
10	7.47	3735.8	0.1916
11	7.79	3895.0	0.0949
12	7.91	3955.5	0.0678
13	7.92	3957.5	0.0594
14	7.92	3960.9	0.0586
15	7.93	3963.3	0.0597
16	7.99	3994.1	0.2031
17	8.24	4116.7	0.0699
18	8.25	4124.5	0.0745
19	8.48	4238.3	0.1460
20	8.89	4444.3	0.0879
21	9.33	4663.1	0.0928

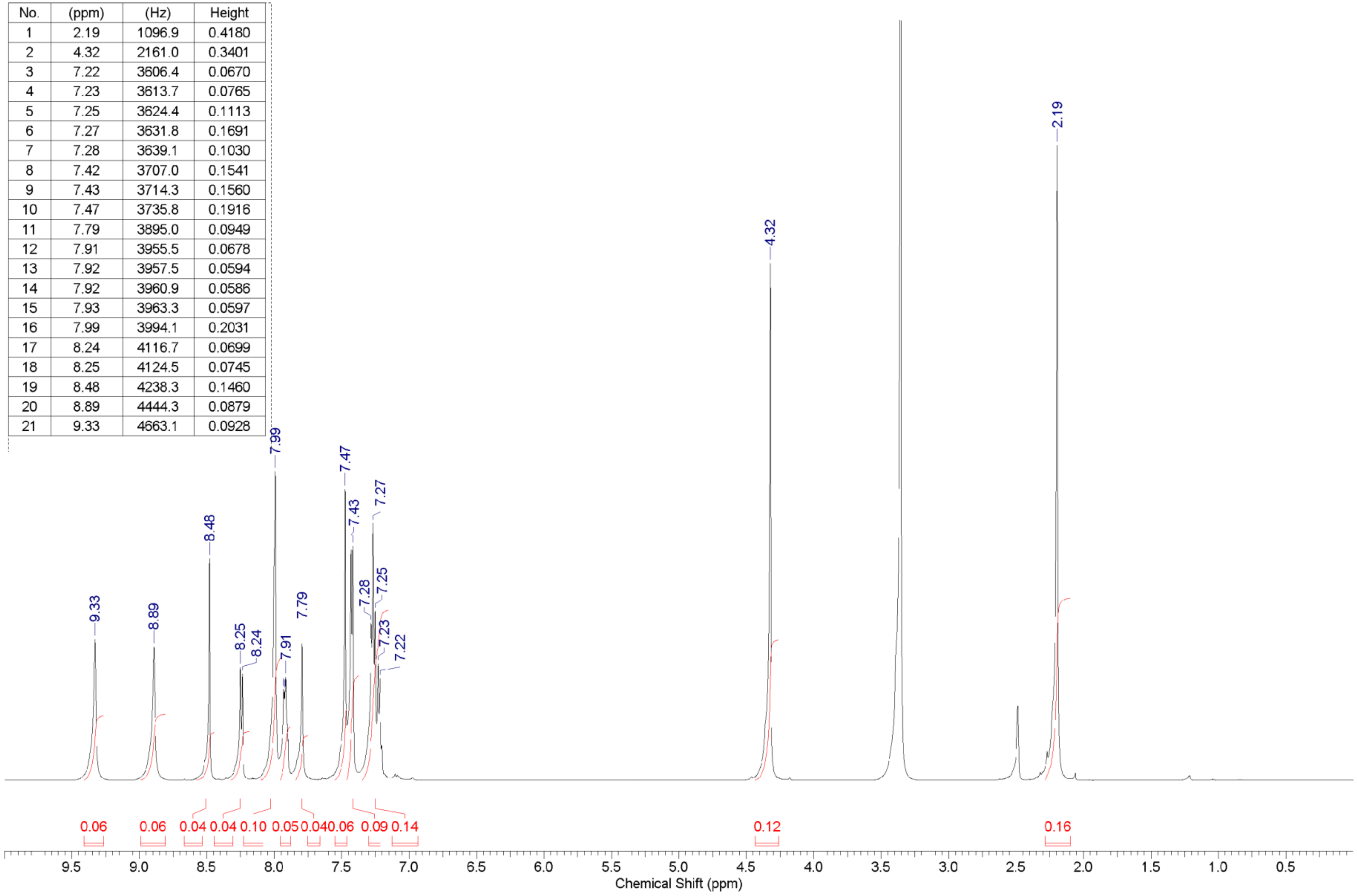

Spectrum 10. ^{13}C-NMR of compound **34** (125 MHz, DMSO-d_6).

No.	(ppm)	(Hz)	Height
1	19.55	2456.9	0.2064
2	37.30	4688.9	0.1724
3	126.69	15923.9	0.1856
4	127.77	16059.6	0.1256
5	127.93	16079.8	0.2081
6	128.10	16100.9	0.1975
7	129.00	16214.6	0.1527
8	129.09	16225.1	0.5482
9	129.86	16322.6	0.5579
10	129.89	16326.6	0.2002
11	131.46	16522.9	0.1783
12	132.84	16696.7	0.1400
13	133.47	16776.2	0.1790
14	135.31	17007.2	0.1129
15	136.84	17199.1	0.1466
16	137.09	17230.7	0.1109
17	138.04	17350.1	0.1191
18	138.34	17388.8	0.1002
19	139.52	17536.3	0.1874
20	155.04	19487.3	0.0844
21	158.02	19861.8	0.1069

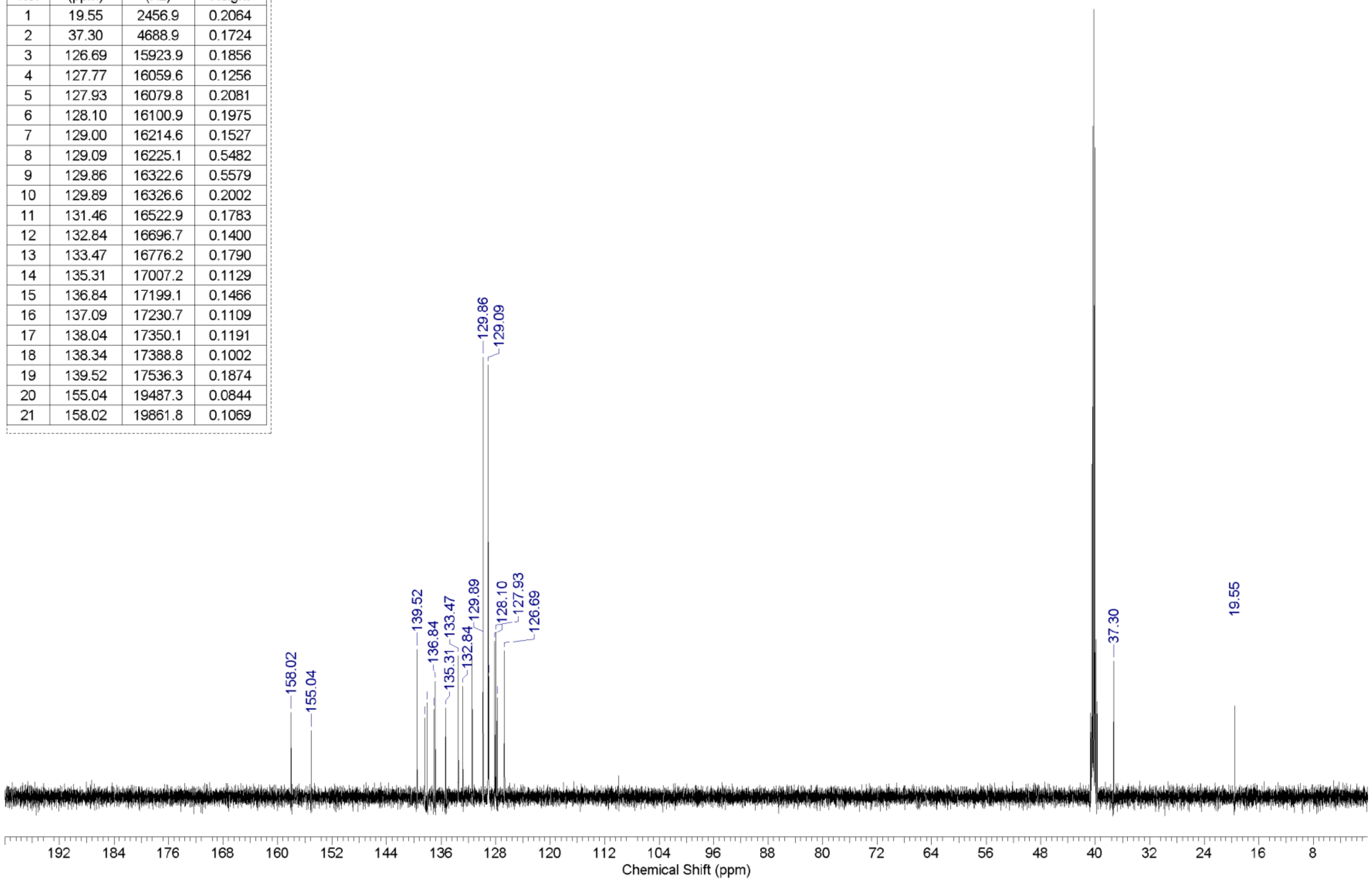

Spectrum 11. ^{1}H-NMR of compound **35** (500 MHz, DMSO-d_6).

No.	(ppm)	(Hz)	Height
1	2.19	1096.0	0.1223
2	4.44	2221.5	0.0920
3	7.49	3743.6	0.0555
4	7.58	3789.0	0.0298
5	7.60	3796.8	0.0518
6	7.64	3816.4	0.0523
7	7.65	3824.7	0.0311
8	7.80	3896.9	0.0273
9	7.90	3947.7	0.0081
10	7.90	3950.1	0.0093
11	7.91	3956.0	0.0182
12	7.93	3961.9	0.0123
13	7.93	3963.8	0.0121
14	7.98	3989.2	0.0097
15	7.99	3994.6	0.0600
16	8.00	4000.0	0.0226
17	8.23	4114.2	0.0212
18	8.25	4122.0	0.0193
19	8.49	4241.2	0.0460
20	8.90	4450.2	0.0113
21	9.35	4674.8	0.0094

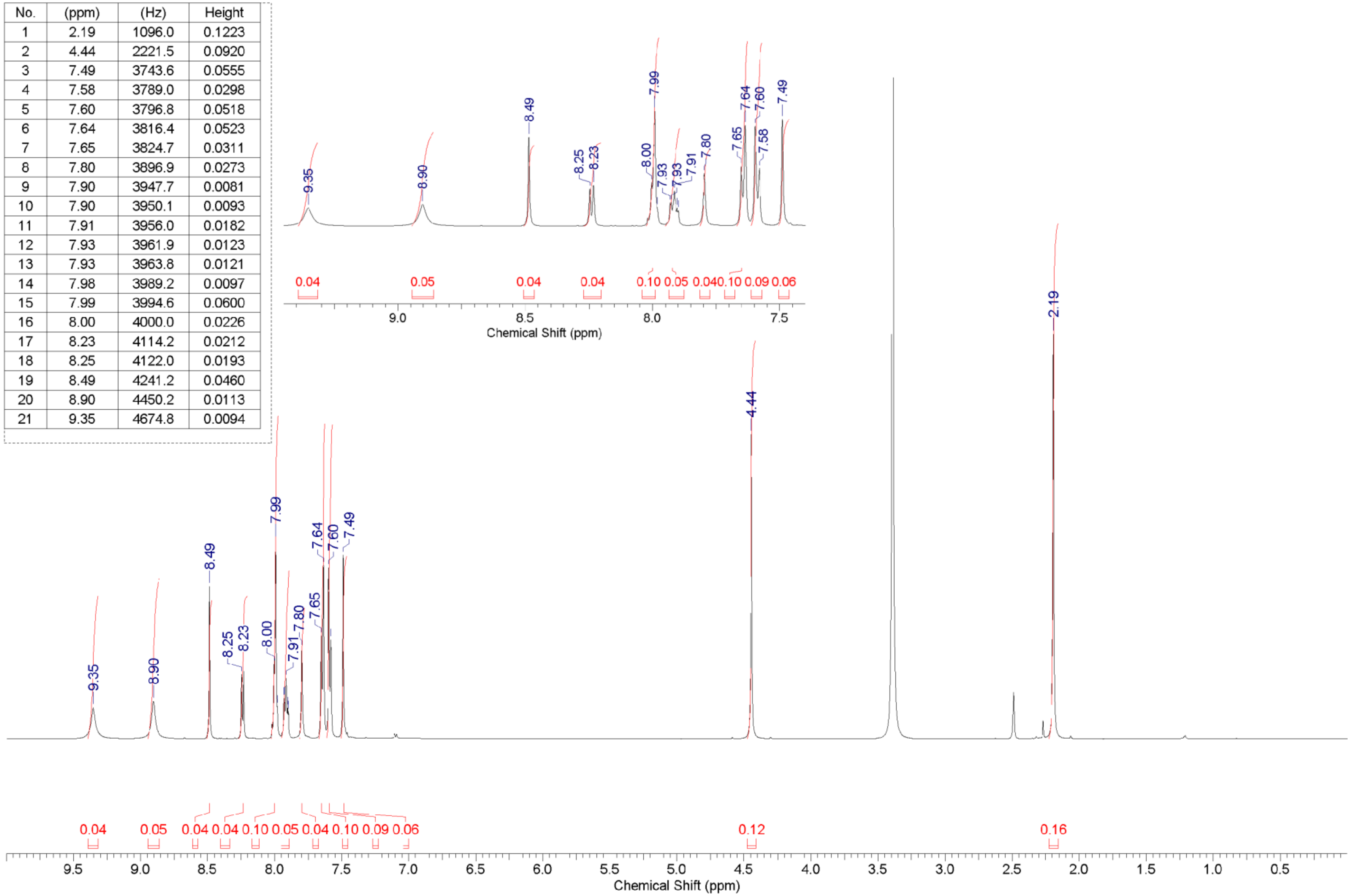

Spectrum 12. ^{13}C-NMR of compound **35** (125 MHz, DMSO-d$_6$).

No.	(ppm)	(Hz)	Height
1	19.55	2457.3	0.2163
2	36.62	4602.4	0.1386
3	125.85	15818.5	0.1728
4	125.88	15822.5	0.1782
5	126.67	15921.7	0.1835
6	127.76	16057.8	0.1325
7	128.11	16102.2	0.1936
8	129.41	16266.0	0.1267
9	129.88	16325.3	0.1431
10	130.56	16410.4	0.5020
11	131.50	16528.1	0.1529
12	133.31	16756.5	0.1354
13	133.49	16778.0	0.1706
14	135.33	17009.4	0.1226
15	136.15	17113.5	0.1224
16	138.08	17355.0	0.1211
17	138.75	17439.7	0.0956
18	139.57	17542.5	0.1764
19	142.21	17874.4	0.0736
20	155.08	19491.7	0.0862
21	158.02	19862.3	0.1170

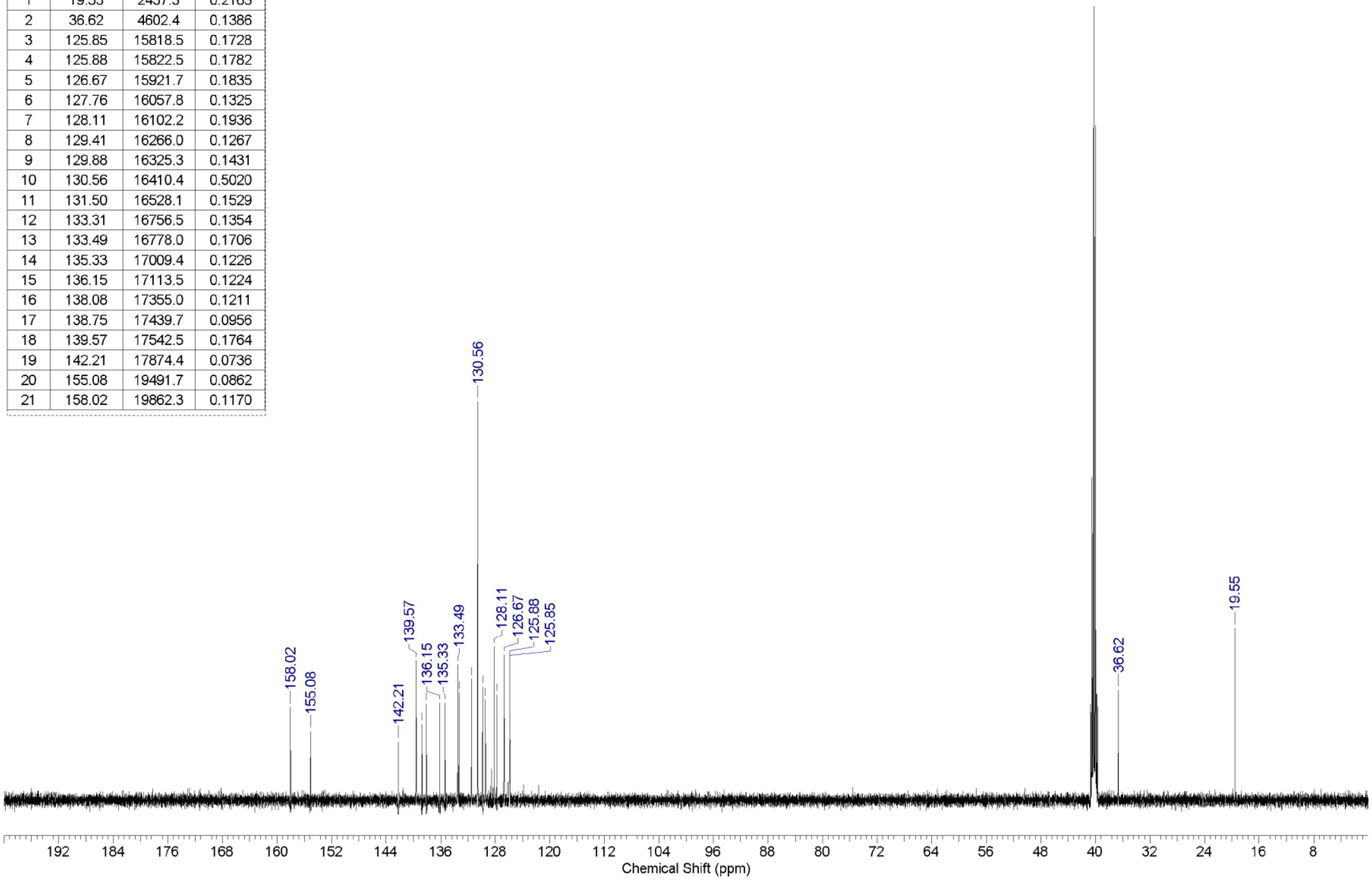

Spectrum 13. ^{1}H-NMR of compound **40** (500 MHz, DMSO-d_6).

No.	(ppm)	(Hz)	Height
1	2.20	1098.4	0.2333
2	4.34	2168.8	0.1937
3	7.30	3646.9	0.0681
4	7.31	3654.7	0.0837
5	7.44	3718.2	0.0997
6	7.46	3726.5	0.0888
7	7.47	3735.8	0.1056
8	7.79	3895.0	0.0577
9	7.92	3958.0	0.0327
10	7.93	3963.8	0.0238
11	8.00	3997.0	0.1139
12	8.24	4116.7	0.0425
13	8.25	4124.5	0.0391
14	8.49	4243.1	0.0894
15	8.89	4442.4	0.0376
16	9.33	4665.5	0.0362

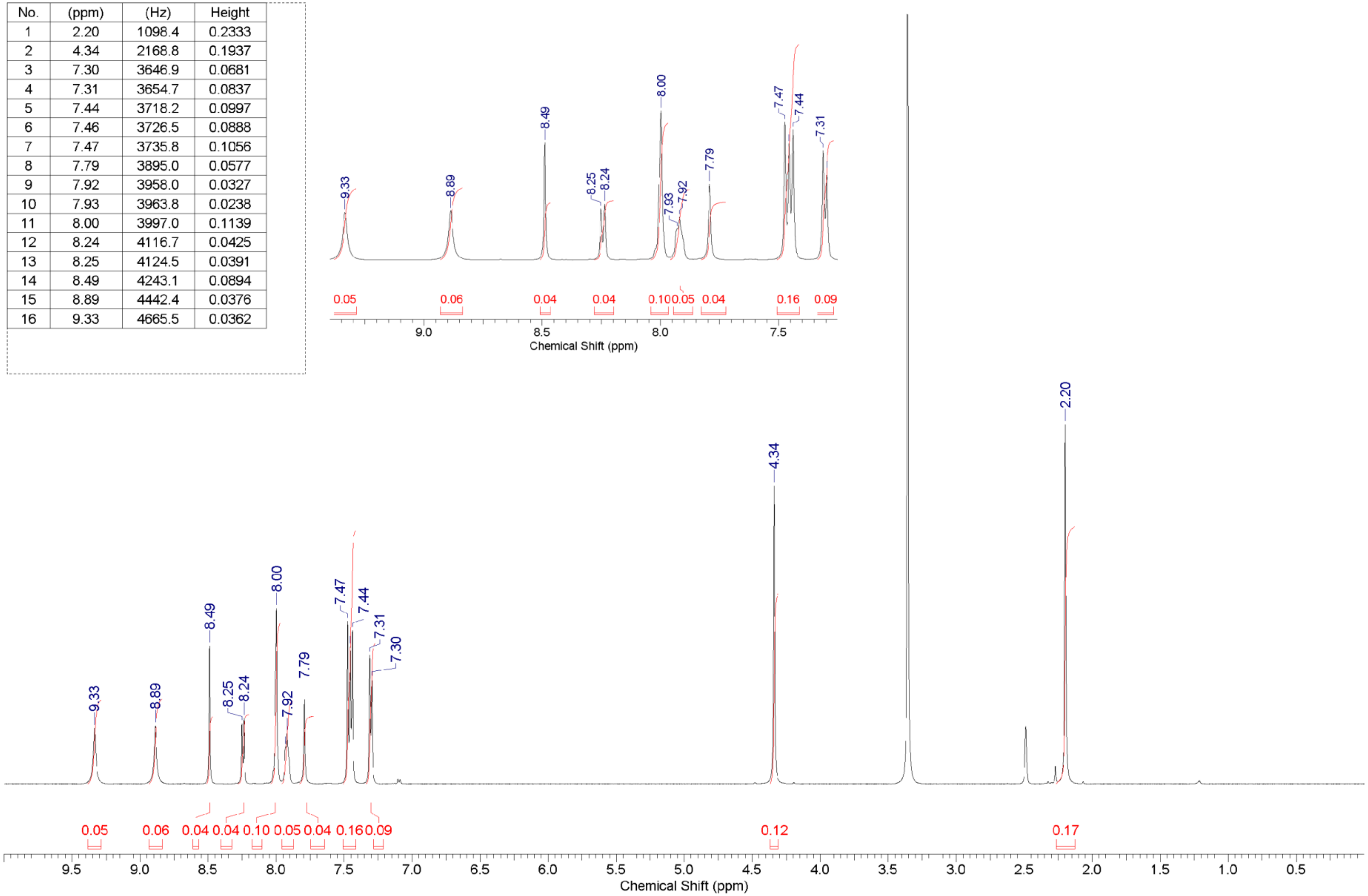

Spectrum 14. ^{13}C-NMR of compound **40** (125 MHz, DMSO-d_6).

No.	(ppm)	(Hz)	Height
1	19.55	2457.8	0.2199
2	36.43	4578.7	0.1657
3	126.69	15923.9	0.1949
4	127.76	16058.7	0.1289
5	128.12	16103.5	0.1857
6	129.02	16216.4	0.5192
7	129.26	16246.7	0.1420
8	129.89	16326.1	0.1489
9	131.48	16525.5	0.1648
10	131.64	16546.1	0.4883
11	132.48	16651.5	0.1448
12	133.10	16729.2	0.1340
13	133.49	16778.9	0.1789
14	135.32	17008.9	0.1367
15	136.13	17109.9	0.1594
16	136.52	17159.1	0.1324
17	138.05	17352.3	0.1315
18	138.59	17419.1	0.1149
19	139.56	17541.1	0.1856
20	155.06	19490.3	0.0923
21	158.03	19862.7	0.1051

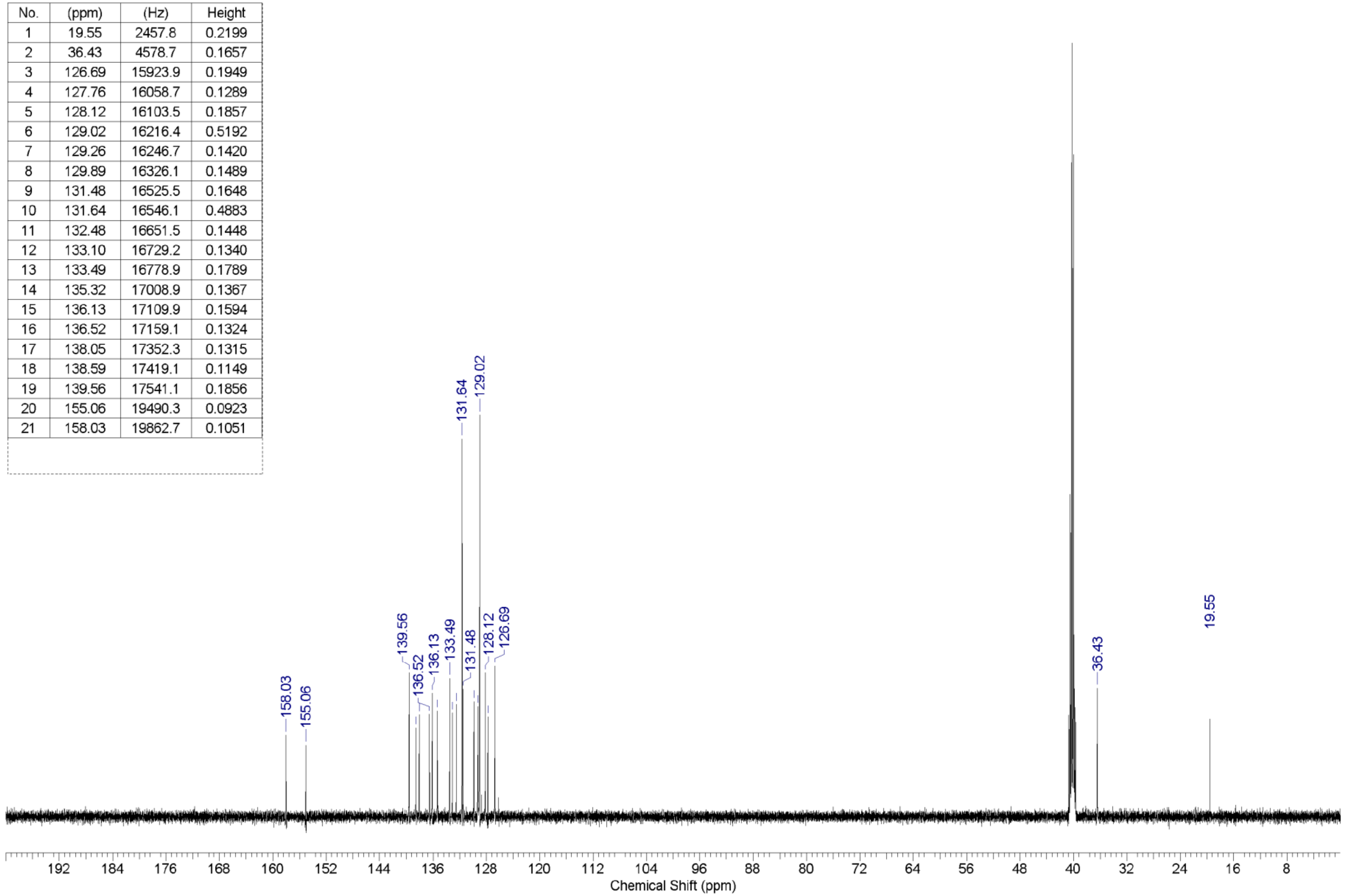

Supplement: Supplementary File 1 [file molecules-19-13704-s001.pdf]
